# Systematic literature review of reproductive outcome associated with residential proximity to polluted sites

**DOI:** 10.1186/s12942-017-0091-y

**Published:** 2017-05-30

**Authors:** Wahida Kihal-Talantikite, Denis Zmirou-Navier, Cindy Padilla, Séverine Deguen

**Affiliations:** 1 LIVE UMR 7362 CNRS (Laboratoire Image Ville Environnement), University of Strasbourg, 3 Rue de l’argonne, 6700 Strasbourg, France; 2 Department of Environmental and Occupational Health, School of Public Health (EHESP), Rennes and Sorbonne Paris Cité, Paris, France; 3 INSERM U1085-IRSET – Research Institute of Environmental and Occupational Health, Rennes, France; 4 Lorraine University, Vandoeuvre-les-Nancy, France; 5 Department of Quantitative Methods in Public Health, School of Public Health (EHESP), Rennes and Sorbonne Paris Cité, Paris, France; 6 Department of Social Epidemiology, Sorbonne Universités, UPMC Univ Paris 06, INSERM, Institut Pierre Louis d’Epidémiologie et de Santé Publique (UMRS 1136), Paris, France

**Keywords:** Systematic review, Residential proximity, Polluted sites, Reproductive outcome, Geographic information systems (GIS)

## Abstract

This study aims to assess the evidence on adverse pregnancy outcome associated with living close to polluted industrial sites, and identify the strengths and weaknesses of published epidemiological studies. A systematic literature search has been performed on all epidemiological studies published in developed countries since 1990, on the association between residential proximity to industrial sites (hazardous waste sites, industrial facilities and landfill sites) and adverse pregnancy outcome (low birth weight, preterm birth, small for gestational age, intrauterine growth retardation, infant mortality, congenital malformation). Based on 41 papers, our review reveals an excess risk of reproductive morbidity. However, no studies show significant excess risk of mortality including fetal death, neonatal or infant mortality and stillbirth. All published studies tend to show an increased risk of congenital abnormalities, yet not all are statistically significant. All but two of these studies revealed an excess risk of low birth weight. Results for preterm birth, small for gestational age and intrauterine growth retardation show the same pattern. There is suggestive evidence from the post-1990 literature that residential proximity to polluted sites (including landfills, hazardous waste sites and industrial facilities) might contribute to adverse reproductive outcomes, especially congenital malformations and low birth weight—though not mortality. This body of evidence has limitations that impede the formulation of firm conclusions, and new, well-focused studies are called for. The review findings suggest that continued strengthening of rules governing industrial emissions as well as industrial waste management and improved land use planning are needed.

## Background

There is growing public and scientific concern regarding the adverse reproductive effects of environmental exposures occurring via three main pathways: contact with ambient air, soil, and drinking water [1, 2]. Most studies published to date have focused on exposure to traffic-related air pollution [3], and several papers have revealed that living near freeways or roadways is associated with toxic effects on both fetus and infant [4–6]. Some studies have examined whether industrial pollution might also alter human health among neighborhood residents [7]. Those who live near polluted sites may be exposed to chemicals released into the air (including off-site migration of gases, dust and chemicals bound to dust, especially during maintenance or transformation operations at the site), as well as through surface or groundwater contamination, or by direct contact with polluted soil. Indeed, these toxicants emanating from polluted sites—including heavy metals, and volatile and other organic compounds—have been reported to affect reproductive outcome around Hazardous Waste Sites (HWS), industrial facilities and landfills [2]. Moreover, the reproductive toxicity of these chemical pollutants has increasingly been documented by toxicological, experimental and animal studies [8]. For instance, some advanced biological mechanisms suggest that heavy metals (cadmium) may affect progesterone production by interfering with steroidogenesis, possibly disturbing endocrine function in pregnant women [9]. These endocrine disruptions constitute a relevant plausible mechanism for an effect on adverse reproductive outcome [10].

Assessment of exposure to emanations from polluted sites is tricky, mainly due to a lack of data on emissions and the cost of acquiring personal exposure data (including biomarkers or other personal data, such as behavioral patterns related to exposure). An alternative way of overcoming these difficulties lies in the use of indirect indicators measuring the proximity of polluted sites, and several types of indicators have been used for this purpose [11–13].

During the 1980s and 1990s, because of growing public awareness and concern about the potential adverse health effects of exposure to chemical contaminants emanating from polluted sites, developed countries drew up environmental laws and waste management guidance policies. For instance, in the United States the *Comprehensive Environmental Response, Compensation, and Liability Act* (CERCLA—also known as Superfund) [14] was set up in order to reduce emissions and protect the environment. This was followed by numerous reforms during the 1990s (e.g. the *Pollution Prevention Act*) [15]. A similar European Union Directive on *Integrated Pollution Prevention and Control* (IPPC) [16] offered waste management guidance—and was transposed into such national legislation as the ICPE (*Installations Classées pour la Protection de l’Environnement*) provision in France [17] and *Pollution Protection and Control* in England and Wales [18]. Implementation of these programs can play an important role in facilitating the cleanup and redevelopment of properties contaminated by hazardous substances. For example, CERCLA affords local government—through the acquisition of contaminated properties—an opportunity to evaluate and assess public safety needs and promote redevelopment projects that will protect and improve the health, environment, and economic well-being of their communities.

Despite improvements in the management of HWS and polluted facilities in developed countries since the 1990s, there is still a question mark as to whether studies may yet reveal excess risks of adverse pregnancy outcome around such sites. Moreover, the fast industrial expansion of emerging countries throughout the world raises the question of the environmental and public health consequences of this development pattern—perhaps its impact will resemble that observed in the 1950s to 1970s in industrialized nations.

A systematic literature review was conducted in order to determine how proximity to environmental hazards impacts the health of neighboring populations, in terms of adverse pregnancy outcome.

The principal objective of the present study is to assess the current evidence on adverse pregnancy outcome associated with living near polluted sites, and to identify the strengths and weaknesses of epidemiological studies published in developed countries since the 1990s, when pollution prevention policies were in effect. An additional objective is to provide more information on the associated health risks with a view to suggesting future directions for research and providing evidence to enhance risk management policies.

## Methods

A systematic literature search was conducted using the Pubmed platform, giving access to the Medline and Academic Search Complete databases, among articles published up to December 2016.

The search strategy followed the Preferred Reporting Items for Systematic reviews and Meta-Analyses (PRISMA) guidelines [19, 20] and was performed with the following keywords found in article titles:

- (industry or industrial or industries or incinerator (s) or polluted site (s) or landfill or hazardous waste (s) or waste site (s) or dumpsites) AND (Fetal or neonatal or infant mortality or miscarriage or stillbirth (s) or infant death or neonatal death or abortion (s) or preterm or prematurity or pregnancy or reproductive or gestational or newborn or birth (s) or birth weight or congenital abnormalities or congenital or congenital abnormality or congenital malformation (s) or small for gestational age or intrauterine growth retardation or birthweight or offspring).

### Selection of studies

**Fig. 1:**
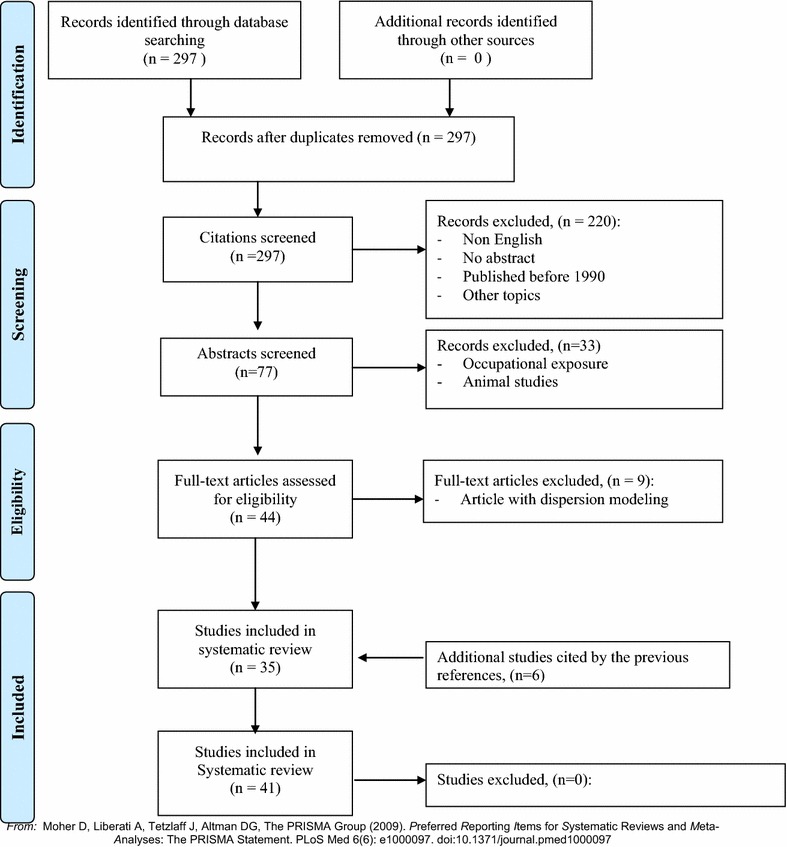
Flow diagram for inclusion and exclusion of studies. From: Moher et al. [20]

At the first step, the inclusion criteria were peer-reviewed papers written in English and articles published after 1990 dealing with the impact of polluted sites on reproductive outcomes without restriction on geographical location (Fig. 1).

Papers presenting non-original studies (e.g. comments, case reports), papers that were published pre-1990 and papers addressing other subjects were ultimately excluded. In all, 77 of the 297 articles published were selected.

At the second step, abstracts of the 77 studies were screened manually by two independent experts (SD and WK, authors of this article); studies were retained if:

(i) they described the indicators measuring the proximity of polluted sites;
(ii) the source of pollution was residential (i.e. non-occupational);
(iii) authors examined a relationship between a human reproductive outcome and a polluted site.

Full manuscripts of the remaining 45 articles (of the 297 initially selected) were thoroughly checked. Because we focused our paper on studies using GIS-based processing functions for spatial exposure assessment, 9 articles using dispersion modeling or interpolation techniques as an exposure assessment method were thus excluded.

Ultimately, a total of 35 articles met the inclusion criteria for the systematic literature review.

Bibliographic reference lists of all included studies were searched manually. Six additional studies cited by the previous references were then included [21–26], resulting in a total of 41 papers that fit the inclusion criteria. Each is reviewed below.

### Extraction data

For each study, the following information was extracted and reported in Tables 1 and 2: general Information (first author’s name, date of study and country of origin), main study characteristics (study design, spatial unit, statistical methods, population definition, database, main findings), participant characteristics (information on confounders), exposure assessment methods and reproductive outcome measures (outcomes classification and definition).

**Table 1:** Literature review of individual studies (cohort and case–control) investigating association between residential proximity to polluted sites and reproductive outcome, order by year of publication

References, year	Design, country	Reproductive outcome	Polluted sites Residential exposure	Confunder factors	Analysis/stratification	Findings
Case–control studies						
Geschwind et al. 1992 [37]	Population-based, case–control study; New York State 1983–1984 USA	All congenital anomalies combined; *Specific defects* Nervous system, Musculoskeletal, system, integument system, oral cleft, digestive system, chromosomal anomalies, syndrome	*Hazardous waste site* Maternal’s exposure defined with exposure risk index that incorporated distance from and the hazard ranking score for each hazardous waste site within 1-mile radius of birth residence	Maternal age, race, education, complication during pregnancy, parity, population density, sex of child	Unconditional linear logistic regression	Results suggested small, statistically significant additional risk for birth defects with maternal residential proximity to toxic waste sites
Shaw et al. 1992^a^ [23]	Population-based, case–control study Five-county San Francisco Bay Area 1983–1985 USA	All congenital anomaly *Specific defects* Central nervous system Eye, Ear, heart/circulatory, respiratory, oral clefts, gastrointestinal, genitourinary, skin, musculoskeletal *Birth outcome* Low birth weight	*Environmental contamination* *Landfill* and *dumpsites* and *HWS* and *Industry…* Exposure defined as Mother’s residence at the time of delivery in a census tract with one or more sites with documented environmental contamination	Maternal race, maternal age, child’s sex, child’s birth order, multiple birth child, gestational age, season of conception, prenatal care	Logistic regression/ linear regression used with LBW	No excess risks found for reduced birth weight or all congenital malformations, combined. However, the results noted elevated risk for heart/ circulatory defects in offspring of mothers who resided in census tracts with sites with evidence of potential human exposure
Sosniak et al. 1994 [30]	Population-based case–control study in 48 states US	All congenital anomalies combined *Birth outcomes* Low and very low birth weight, infant deaths, fetal deaths	*NPL site* Mother exposure defined as a distance of 1 mile or less from nearest NPL site from zip code centroids of maternal residences at delivery	Prenatal care, smoking, drinking and illicit drug use status, working history. Education, income, age at pregnancy, and sex of the child	Univariate and multivariate analyses were performed	Maternal residential proximity to NPL sites not associated with adverse pregnancy outcomes including: Congenital anomalies, low and very low birth weight, infant deaths, fetal deaths
Goldberg et al. 1995 [54]	Population-based, case–control study Montreal (1979–1989) Canada	LBW, VLBW Preterm births, Small-for-gestational age (less than or equal to the third percentile weight for gestational age)	*Landfill site* Defined three exposure zones representing areas proximal and distal to a municipal soil id waste landfill site. High exposure zone divided into two subzones to account for prevailing winds	Mother’s age, education level, marital status, usual language spoken, season of birth, sex of the newborn	Unconditional logistic regression	Among births to mothers who resided adjacent to the landfill: Significant elevated risk of LBW and no-significant elevated risk of small for gestational age. But no significant positive association were observe for PTB or for VLBW
Croen et al. 1997^a^ [35]	Population-based, case–control study California 1989–1991 US	*Specific defects* NDt, conotruncal heart, and oral cleft defects	*Hazardous waste site* Maternal exposure defined as living during periconceptional period in either: In census tract contained one or mmore waste site—within 1 mile or less of one or more sites	Sex of baby, Maternal age Race/ethnicity, Maternal education, Family income Periconceptional employment status Alcohol use, Smoking Vitamin use, Education	Multivariate analyses using unconditional logistic regression	No increased risks for congenital defects for a maternal residence in a census tract with one or more waste sites, but some association was noted between a maternal residence within ¼ mile of an NPL site and risk for NTD and conotruncal heart defects in offspring
Dolk et al. 1998 [52]	Population-based case–control study Belgium, Denmark, France, Italy, UK	Non-chromosomal congenital anomalies *Specific defects* NDt, cardiac septa, Anomalies of great arteries and veins, central nervous system, oral defect…	*Landfill site* Within each study area, a 0–3 km “proximate” zone was defined around each landfill site with hazardous waste. This zone was compared with a 3–7 km “distant” zone.	Socioeconomic status and maternal age	Logistic and related binomial regression models were used	Results indicated significant small excess risk of non-chromosomal defects in offspring among women who lived near hazardous waste landfill sites Elevated odds ratios were also found for specific defects
Marshall et al. 1997^a^ [38]	Population-based, case–control study 18 counties in New York State, 1983–1986 USA	*Specific defects* Central nervous system and musculoskeletal system defects	*HWS* and *industrial sites* Mother’s exposure defined as maternal living at delivery within 1 mile of industrial facilities that release specific air emissions (TRI) or to waste sites with specific contaminants	prenatal care, mother’s education, mother’s age, mother’s race, total previous births, trimester prenatal care initiated child’s sex, urban–rural status (Population density)	Unconditional logistic regression model	No increased risk noted between women living in areas with a medium or high probability of exposure to chemicals from hazardous waste sites and CNS and musculoskeletal birth defects in offspring; however, association seen between living in close proximity to industrial facilities with emissions of soil vents or metals and CNS defect
Orr et al. 2002^a^ [12]	Case–control study 1983–1988 California (24 counties)	All birth defects combined *Specific defects* NDT, MUS defects, CNS defects, integumental defects, heart or circulatory defectsoral cleft defects, and conotruncal heart defects	*Waste site* Exposed defined as maternal address at child’s birth in census tract with one or more National Priority List (NPL) hazardous waste sites	sex, Maternal age, Racial/ethnic group, Prenatal care Birth outcome	Logistic regression model	Strongest association observed between a maternal residence in a census tract with one or more NPL sites and birth defects in offspring
Vriljheld et al. 2002^a^ [1]	Population-based case–control study Belgium, Denmark, France, Italy, UK	chromosomal and Non-chromosomal anomaly *Specific defects* NDT, cardiac septal defects, malformations of the great arteries and veins	*Landfill site* Within each study area, a 0–3 km “proximate” zone was defined around each landfill site with hazardous waste This zone was compared with a 3–7 km “distant” zone	Maternal age and socioeconomic status	Logistic regression models were used	The result noted that there is little evidence for a relation between risk of congenital anomaly in proximate relative to distant zones and hazard potential of landfill sites as classified by the expert panel
Vriljheld et al. 2002 [51]	Population-based case–control study Belgium, Denmark, France, Italy, UK	Chromosomal congenital anomalies *Specific defect* Down’s syndrome, non-Down’s syndrome	*Landfill site* Within each study area, a 0–3 km “proximate” zone was defined around each landfill site with hazardous waste This zone was compared with a 3–7 km “distant” zone	Adjusted for maternal age and socioeconomic status, study area, year of birth	Logistic and related binomial regression models were used	An increased risk of chromosomal anomalies with a maternal residence near hazardous waste landfill sites was noted. Whereas, risk did not decline consistently with increasing distance from sites
Boyle et al. 2004 [41]	Population-based cohort and case–control studies; Eastern Region of Ireland births, 1986–1990 Great Britain	All congenital anomalies combined	*Landfill site* Municipal landfill sites within 3 km (and other distances) of district electoral divisions; distance of case and control addresses from landfill sites			Living near a municipal landfill site was not found a risk factor for congenital malformations
Malik et al. 2004 [31]	Population-based case–control study Dallas County, 1979–1984 USA	Live births diagnosed with congenital heart disease at any age	*Hazardous waste site* Mothers’ exposure defined as residence at delivery within ¼ and 1 mile of hazardous waste site	Stratification by CHD category	Chi-square and Mantel Haenszel analysis used to estimate odds ratios	Small, but statistically significant, additional risk (20%)for congenital heart disease among offspring of women who lived near a hazardous waste site (1 mile)
Yauck et al. 2004 [22]	Population-based case–control study; Milwaukee, Wisconsin 1997–1999 USA	Congenital heart defect (CHD) among older women	*HWS* and *Industrial facilities* Mother’s exposure defined as address at delivery within 1.32 miles of waste sites and industrial facilities with emissions of trichloroethylene	Race/ethnicity, cigarette use, prenatal care received, month of pregnancy prenatal care began, pregnancy-associated hypertension, gestational diabetes	Backward stepwise Logistic regression	Maternal residential proximity to waste sites and industries with TCE emissions associated with CHD in offspring of older but not younger women
Brender et al. 2006^a^ [32]	Population- based case–control study Texas, 1996–2000 US	*Specific defects* Live births and fetal deaths with cleft palate without cleft lip; cleft lip without or with cleft palate; isolated oral cleft	*HWS* and *Industries sites* Residence at delivery (and during the periconceptional period) within 1 mile of NPL or state hazardous waste site and/or within 1 mile of industries	Maternal race/ethnicity, education, and tobacco use	Logistic regression used to obtain odds ratios	Maternal residential proximity to industries might be associated with oral clefts in births to older mothers (>*35 years)*
Mueller et al. 2007^a^ [40]	Population-based case–control study in Washington 1987–2001 USA	All fetal death 2 timing of fetal death: −<28 weeks (early) −≥28 weeks gestation (late) excluded those with gestational age <20 weeks	*Hazardous waste site* Measured straight-line distances in miles between the mother’s residence at the time delivery and the nearest hazardous waste site	Maternal age, prenatal smoking status, and number of prior pregnancies	Stratified analyses using Mantel-Haenszel risk estimators multivariable logistic regression	Fetal death not associated with maternal residential proximity to hazardous waste sites
Kuehn et al. 2007^a^ [34]	Population-based case–control study in Washington State, 1987–2001 US	Any congenital malformation *Specific defects* CNS, GI, Heart, Reproductive/urogenital, Musculoskeletal, Ears/eyes/nose/Respiratory/circulatory, Chromosomal Skin, Other Midline	*Hazardous Waste site* Distance of women’s residence at time birth from hazardous waste sites; proximity defined as various distances up to 5 miles;	Maternal and paternal age, maternal smoking and alcohol consumption, parity, gravidity, prior fetal death, race/ethnicity, maternal education, county of residence, medical insurance status, marital status, parental employment urban vs. rural residence, census tract median income, and census tract population density	Multivariable logistic regression	An increased risk of congenital malformations among offspring of women living in close proximity of hazardous waste sites; Moreover, associations for malformations stronger with sites in urban areas than in rural areas
Suarez et al. 2007^a^ [39]	Population-based case–control study in Texas, 1996–2000 USA	*Specific defect* Neural tube defects	HWS and industrial site Mother exposure defined as residence at delivery within 1 mile of state or NPL hazardous waste site or within 1 mile of industries with reported air emissions of chemicals	Maternal age, race/ethnicity, education, and maternal and paternal occupational exposures	Logistic regression used to calculate ORs	No excess risk noted for NTDs in offspring among women living near hazardous waste sites; however, close proximity to industrial facilities with chemical air emissions associated with NTDs in several subgroups
Brender et al. 2008^a^ [33]	Population-based case–control study in Texas, 1996–2000 USA	Chromosomal anomalies (combined) and categorized into nine categories based on BPA codes	*HWS* and *Industrial site* Mother exposure defined as residence at delivery within 1 mile of industries with reported air emissions of chemicals or residence at delivery within 1 mile of state or NPL hazardous waste site	Year of birth, Infant sex, public health region of maternal residence maternal age education and race/ethnicity	Unconditional logistic regression and exact logistic regression	Maternal residence within 1 mile of a hazardous any waste site or of an industrial facilities was not associated with chromosomal anomalies in offspring. However, results suggested some relation between residential proximity to specific type of industries and specific defects
Langlois et al. 2009^a^ [13]	Population-based case–control study of Texas 1996–2000 USA	Conotruncal heart defects with and without chromosomal anomalies and truncus arteriosus, transposition of the great vessels, and tetralogy of Fallot separately	HWS and industrial site Mother exposure defined as maternal address at delivery within 1 mile to hazardous waste sites and industrial facilities (Toxic Release Inventory (TRI))	Maternal age, race/ethnicity, education, maternal and paternal occupation and employment industry	Logistic regression / exact logistic regression used to calculate ORs	Proximity to waste sites or industrial facilities not associated with conotruncal heart defects, while result noted truncus arteriosus associated with a maternal residence within 1 mile of any waste site and with NPL sites
*Cohort studies*						
Dodds et al. 2001 [55]	Population-based cohort study Nova Scotia, Canada 1988–1998	All anomalies combined *Specific defects* NDT, cardiovascular, Genito-urinary, Musculoskeletal, Ear, Eyes, Nose, Throat, Chromosomal *Birth outcome* LBW, PTB, intrauterine growth retardation (IUGR)	*Hazardous waste site* Rates for malformations and other adverse pregnancy outcomes compared by maternal address at the time of delivery in Sydney (site of hazardous waste site), Nova Scotia, and Cape Breton County (excluding Sydney)	Maternal age, maternal smoking, parity	Logistic regression models	Small statistically significant increase in rate of major congenital malformations in community with a hazardous waste site
Dummer et al. 2003 [44]	Retrospective cohort study Cumbria (northwest England), 1956–93 United Kingdom	Deaths from congenital anomaly (ICD 740–749): *(all NDT, CHD, other congenital anomalies)* *Birth outcome* Stillbirth occurring (after 28 weeks gestation) Neonatal death (within the first four weeks of life)	*Incinerators* *Crematoriums* Distances of maternal address at child’s birth from incinerators and crematoriums	Year of birth, social class, birth order, multiple births yes/no	Multivariate logistic regression Stratified by time period	*Incinerators.* The risk of stillbirth and neonatal death was not significantly increased closer to incinerators, but the risk of lethal congenital anomaly was significantly higher *Crematoriums.* Increased risk of anencephaly, other congenital anomalies, and stillbirth closer to crematoriums
Dummer et al. 2003 [43]	Retrospective cohort study Cumbria (northwest England), 1956–93 United Kingdom	Deaths from congenital anomaly (ICD 740–749): *(all NDT, CHD, other congenital anomalies)* *Birth outcome* Stillbirth occurring (after 28 weeks gestation) Neonatal death (within the first four weeks of life)	*Industrial site* Distances of maternal address at child’s birth from industrial site	Year of birth, social class, birth order, multiple births yes/no	Multivariate logistic regression Stratified by time period	There were no significantly increased risks for stillbirth or neonatal death in relation to proximity to industrial sites The only significant result was an increased risk of deaths from congenital heart defects closer to industrial sites in the 1983–93-time period
Dummer et al. 2003^a^ [42]	Retrospective cohort study Cumbria (northwest England), 1956–93 United Kingdom	Deaths from congenital anomaly (ICD 740–749): *(all NDT, CHD, other congenital anomalies)* *Birth outcome* Stillbirth occurring (after 28 weeks gestation) Neonatal death (within the first four weeks of life)	*Landfill site* Distances of zip code of maternal address at child’s birth from Landfill classified by the type of waste treated	Year of birth, social class, birth order, multiple births yes/no	Multivariate logistic regression Stratified by time period	There was no increased risk of any other lethal adverse pregnancy outcome associated with residence near the landfills site. However, a small significantly increased risk of death from “Other congenital anomalies of nervous system” was found in children of mothers living near domestic waste landfill sites
Morgan et al. 2004 [50]	Retrospective cohort study of singleton live births in England, 1986–1999 United Kingdom	Low birth weight births	*Landfill site* Mother’s exposure defined as residence at delivery within 3 km of a landfill; for all study areas pooled, defined 1-km distance bands with 6–7 km as baseline	Sex, quintiles of Carstairs deprivation index, year of birth	Logistic regression used to estimated odds ratios	A small and not statistical significant increase in LBW risk associated with a maternal residence near landfill sites in England. Whereas a significant increase excess risk of congenital malformation associated with a maternal residence near the same sites
Tango et al. 2004 [56]	Retrospective cohort of Japan (1997–98)	Infant, neonatal, and fetal deaths due to congenital malformations (all combined), sex ratio, LBW: (<2500 g), VLBW (<1500 g), Neonatal deaths (under four weeks of age), early neonatal deaths (under one week of age), Infant deaths (under 1 year of age), Fetal deaths, spontaneous fetal deaths (after 12th week of gestation) spontaneous fetal deaths with CA	Incinerators The study area was defined as circles of radius 10 km from the MSW incinerators Area close to the MSW incinerator defined as to be 0–2 km	Maternal age, Gestational age Birth weight, total previous deliveries, Past experience of fetal deaths, type of paternal occupation	Stone’s unconditional test and tango’s conditional test for decline in risk (O/E ratio) with distance from the incinerator. P-values of these unconditional and conditional tests were calculated using 9999 Monte Carlo simulations	None of adverse reproductive outcomes showed statistically significant excess for all the zones A statistically significant peak-decline in risk with distance from the incinerators up to 10 km was found for infant death and Infant death with all Congenital anomaly A decline in risk with distance from the incinerators was observed for spontaneous fetal deaths with Congenital anomaly
Palmer et al. 2005 [46]	Population-based cohort in Wales 1983–1997 Great Britain	All Congenital anomalies *Specific defect* Chromosomal anomalies, cardiovascular defects, and abdominal wall defects	*Landfill* Exposure defined as birth living within 2 km of the centroid of landfill sites, before and after opening of the sites, with referent group living at least 4 km away from these sites	Maternal age, hospital of birth, year of birth, deprivation, sex of baby	Expected rates were calculated from a logistic regression model	Increased risk of congenital anomalies after the opening of landfill sites from 1983–1997 but increase did not persist during 1998–2000
Gilbreath et al. 2006 [29]	Retrospective cohort study Alaska Native villages, 1997–2001	LBW (>1500 to <2500) VLBW (<1500 g), PTB (<37 weeks) IUGR (at least 37 weeks’ gestation and <2500 g	*Dumpsites* Hazard ranking of the dumpsite of the village that was indicated on the birth certificate Residence in villages with open low, intermediate and high hazard dumpsites	Gender, interpregnancy interval, parity, adequacy of prenatal care, smoking status, alcohol intake, race, mother’s age and education, health care options, piped water, and missing values	Logistic regression	Infants from mothers in villages with intermediate and high hazard dumpsites had a higher proportion of LBW and suffered from intrauterine growth retardation Slighty reduced risks for preterm birth in mothers from intermediate hazard villages
Gilbreath and Kass 2006 [36]	retrospective cohort study of live births and fetal deaths Alaska Native villages, 1997–2001 USA	Fetal deaths (>20 weeks of gestation), Neonatal deaths *Specific defects* Congenital anomalies grouped into five categories including central nervous system, circulatory and respiratory, gastrointestinal, urogenital, and musculoskeletal or integumentary defects	*Dumpsites* Hazard ranking of the dumpsite of the village that was indicated on the birth certificate Mother’s exposure defined as residence in villages with open low, intermediate and high hazard dumpsites	Gender, interpregnancy interval, parity, adequacy of prenatal care, smoking status, alcohol intake, race, mother’s age and education, healthcare options, piped water	Poisson regression used to model the natural log of the incidence rates	No significant excess risk was found for fetal deaths, neonatal deaths, or congenital anomalies with a maternal residence in Alaska Native villages with higher hazard dumpsites; except for one group of congenital anomaly

^a^Authors study specific sites classified by either substance class or reported air emissions of chemicals, or types of contaminants present and media contaminated or with respect to human exposure potential, or contaminated environmental media, and chemical contaminants present

**Table 2:** Literature review of ecological studies investigating association between residential proximity to polluted sites and reproductive outcome, order by year of publication

Reference, year,	Design,	Reproductive Outcome	Polluted sites Residential exposure	Confunder factors	Analysis/stratification	Findings
	Country					
Berry et al. 1997 [27]	Birth certificate-based study Philadelphia 1961–1985 USA	LBW Preterm birth (<37 weeks)	*Landfill site* Mother’s exposure defined as living closest to landfill (the only neighborhood adjacent to the landfill and lake) or 1.0 km was extended from the perimeter of the landfill	Potential risk factor: Maternal age, education, parity, number of previous stillbirths, poor parental care, sex of the child	Logistic regression	Among term births (37–44 weeks) Parent living closest to landfill (the neighborhood immediately adjacent to the landfill) had statistically significant higher proportion of LBW and twice the risk of being born preterm
Bhopal et al. 1999 [48]	Ecologic study in Teesside and Sunderland, 1986–1993 United Kingdom	All congenital abnormalities (excluding isolated minor congenital abnormalities), *Birth outcomes* low birth weight, stillbirth, sex ratio	*Petrochimical industries* Residential proximity to major steel and petrochemical industries in Teesside divided into three zones based on distance with Sunderland serving as the reference population	–	Unclear	No excess risk of adverse pregnancy outcomes associated with living near major steel and petrochemical industries, exception of low birth weight
Eizaguirr et al. 2000 [25]	Population-based descriptive geographical study during 1982–1989 in Glasgow and nearby areas United Kingdom	All congenital anomalies combined	*HWS* A 10 km circle centred around former site of factory site designed as study areas and divided into 2 km area containing site and 8 concentric rings around it, each 1 km wide	Carstairs deprivation category	Poisson regressions	Findings suggest that any possible teratogenicity caused by chromium is not apparent. The risk of congenital anomaly is lowest in the area within the first 2 km, and the risk peaks between 2 and 4 km
Fielder et al. 2000 [21]	Ecologic study of population in South Wales, 1983–1996 United Kingdom	All congenital anomalies combined *Specific defect* Anomalies of the abdominal wall, *Birth outcome* LBW Spontaneous abortion	*Landfill site* The exposed population defined as residents living in the five electoral wards within 3 km of the landfill site	Townsend index	Poisson cumulative probabilities were calculated Comparaison between exposed population (within 3 km) and the rest unexposed population	Increased risk for congenital malformations in births among residents living near the site both before opening and after opening There were no consistent differences in proportion of low birth weight infants or spontaneous abortion between the two populations
Elliott et al. 2001 [45]	Ecologic study Great Britain, 1983–1998	All congenital anomalies combined; *Specific defects* NDT, cardiovascular, and abdominal wall defects; hypospadias and epispadias; surgical correction of hypospadias and epispadias; surgical correction of gastroschisis and exomphalos; *Birth ouctome* Still births; LBW, VLBW	*Landfill site* Mother’s exposure defined as residential postcodes within the 2 km buffer zone around site	Year of birth, administrative region, sex of birth, deprivation	Model prediction from poisson regression of data from the reference area to provide standard rates	Small excess risk of congenital anomalies and low and very low birth weight in populations living within 2 km of landfill sites. However, no significant positive association was observe for stillbirth
Baibergenova et al. 2003 [28]	Ecologic study of New York (excluding New York City) (1994–2000) US	VLBW, LBW	*PCB-contaminated site* Exposure defined as maternal residence at birth in a zip code that contained or was adjacent to a PCB-contaminated site	Sex of the baby, race of the mother, mother’s age, father’s age, mother’s educational level, parents annual per capita income, Medicaid/self-paid births, Maternal weight Single motherhood Maternal smoking	Multiple logistic models Stratification by sex of the baby	Slight association noted for risk of low birth weight in male births and maternal residence in zip code with one or more waste sites contaminated with PCBs. But, no relation between PCB zip codes and very low-birth-weight infants for either sex
Morris et al. 2003 [47]	Ecologic study in Scotland (1982–1997) Great Britain	All congenital anomalies *Specific defect.* NTD, cardiovascular, and abdominal wall defects; hypospadias and epispadias; surgical correction of hypospadias and epispadias; surgical correction of gastroschisis and exomphalos; *Birth outcome.* LBW; LBW, Stillbirths	*Landfill site* Mother exposure defined as residential postcodes within 2 km buffer zone around each site	Year of birth, sex deprivation	Model prediction from poisson regression	No statistically significant excess risks of adverse pregnancy outcomes (LBW, stillbirth, Congenital anomalies) detected in population living within 2 km of a hazardous waste site
Cresswell et al. 2003 [24]	Ecologic study in city of New Castle upon Tyne, 85–99 United Kingdom	*Specific defects* Chromosomal and non-chromosomal defects	*Waste combustion plant* Mother’s exposure defined as residence within 3 km of Byker waste combustion plant	ED-level deprivation Not able to adjust for other characteristics	Poisson regressions used to estimate Rate ratios for congenital anomaly	Little evidence of relation between prevalence of congenital malformations and residence near waste combustion plant
Kloppenbor et al. 2005 [26]	Ecological study in Denmark, 1997–2001 Denmark	All congenital anomalies combined *Specific defects* The nervous or cardiovascular systems in live births	*Landfill* Three buffer zones: 0–2 (proximal zone), 2–4 (middle zone), and 4–6 km (distal zone) was constructed surrounding waste landfill sites	–	The risk rate (RR) was calculated by dividing the sum of congenital anomaly (or specific defects) by total proximal sum of births	No association found between maternal residential proximity to landfills and all congenital malformations combined or of the nervous system. However, the result noted small excess risk for anomalies of the cardiovascular system
Bentov et al. 2006 [57]	Ecologic study of live births and stillbirths Beer-Sheva subdistrict 1995–2000 Israel	Major congenital malformations combined *Specific defects* Central nervous system, chromosomal anomalies and other major congenital malformations	*Industrial park* Distance of localities from regional industrial park and predominant wind direction		Calculation of rateby dividing the number of newborns born with birth defect by the number of deliveries	Residential proximity to industrial park associated with increased rates of major congenital malformations among Bedouin populations
Jarup et al. 2007^a^ [49]	Ecologic study of England and Wales 1989–1998 Great Britain	*Specific defects* Down syndrome	*Landfill site* Mother exposure defined as an residential address within 2-km zone of a landfill site	Maternal age Urban–rural status, Carstairs’ index of deprivation	Regression modelling within a Bayesian framework	No excess risk of Down syndrome noted in populations living within 2 km of a landfill site, regardless of site type
Elliott et al. 2009^a^ [11]	Ecologic study in England, 1983–1998 Great Britain	All congenital anomaly combined *Specific defects* hypospadias and epispadias, cardiovascular defects, NDt, and abdominal wall defects	*Landfill site* Divided England into a grid of 5 × 5 km squares in which births in each square were classified in terms of its proximity to a landfill site 1 year previously (<2 km, 2+ km) to an index	Carstairs score Presence or absence of a local congenital anomalies register Maternal age % industrial land % urban land	Bayesian hierarchical logistic regression models used with random effects to obtain odds ratios	Significant weak associations observed between risk of all anomalies combined and specific defects and geographic density of only special wastes sites at the level of 5 × 5 grid squares
Castello et al. 2013 [53]	Ecologic study (2004–2008) Spain	VPTB, <33 weeks MPTB, 33–36 weeks VLBW, <1500 g MLBW, 1500–2499 g SGA, birth weight below the national 10th percentile for babies of the same gender and gestational age	*Industries site* Mothers’ exposure to industrial pollution was estimated by taking the distance from the administrative center of municipality of residence to the pollution source	% adolescent mothers, % mature mothers, % immigrant mothers coming from countries with low income, % mothers who were illiterate mothers or did not complete primary school education, % mothers developing manual work, Population size, habitability index, unemployment rate, average socioeconomic level, % mono-parental families, number of vehicles per household	A Besag, York, and Mollié (BYM) model was fitted for each combination of the 5 outcomes and 24 industrial activity groups	Association between residential proximity to certain types of pollutant industrial facilities and increased risk of some adverse birth outcomes Excess risk of MLBW seemed to be associated with residential proximity to facilities from most of the industrial groups

^a^Authors study specific sites classified by either substance class or reported air emissions of chemicals, or types of contaminants present and media contaminated or with respect to human exposure potential, or contaminated environmental media, and chemical contaminants present

The present paper comprises 5 sections. First section: “Bibliographic material” presents an outline of the different study designs, followed by the various categories of reproductive outcome related to residential proximity to polluted sites, and finally the environmental contaminations that were explored. The findings of this section are summarized in Tables 1, 2 and 3. Second section: GIS methodology presents an overview of analytical methods used to assess residential proximity to polluted sites using approaches based on GIS and according to type of polluted sites. The findings of this section are summarized in Table 4. Third section: Current evidence on the possible effects of proximity to polluted sites addresses the question of whether or not proximity to polluted sites can affect reproductive outcome. Fourth section is a discussion of the general methodological issues relevant to epidemiological investigation of the effects of proximity to polluted sites on reproductive outcome. Fifth section offers conclusions and recommendations for improving future research on these issues.

## Bibliographic material

Tables 1 and 2 provides the characteristics of all the studies reviewed, by year of publication, type of study design, pregnancy outcome, exposure assessment and major findings and conclusions.

### Study location

Most studies were conducted in the United States (18) [12, 13, 22, 23, 27–40] and the UK (14) [11, 21, 24, 25, 41–50]. We also found five studies conducted in continental Europe [1, 26, 51–53], two in Canada [54, 55] and two in Asia [56, 57] investigating whether living near a polluted site increases the risk of adverse reproductive outcome. Contrasted descriptions were revealed in term of study location, period of publication, outcomes and polluted sites of interest according to the design of the study (more details in “Study design” section).

### Design and database

With the exception of a single descriptive geographical study [25] and eleven ecological studies [11, 21, 24, 27, 28, 45, 47–49, 53, 57], all papers analysed individual data, including ten cohort studies [29, 36, 42–44, 46, 50, 55, 56] and 18 case–control studies [1, 12, 13, 22, 23, 30–35, 37–40, 51, 52, 54]. Most databases were drawn from either congenital registers or birth certificates (see Table 3).

**Table 3:** Summary of reproductive outcomes related to the polluted sites (order by outcome)

Outcomes	Polluted site	Study design	Population study	Database study	Methods	References
Birth outcome (LBW/PTB)						
LBW	Landfill	Ecologic study	All live births	National birth data-based study	Poisson regression model	Elliot et al. 2001 [45]
			All births in Scotland	National birth data-based study	Model prediction from poisson regression	Morris et al. 2003 [47]
			All births	Register of the office for national statistics	Poisson cumulative probabilities were calculated	Field et al. 2000 [21]
		Birth certificate-based study	All births	Birth certificate	Logistic regression model	Berry et al. 1997 [27]
		Case–control study	All live births to residents on Island of Montreal, 1979–1989 (excluded multiple birth and births to parous mothers)	Birth registration	Unconditional logistic regression	Goldbrg et al. 1995 [54]
		Retrospective cohort study	All singleton live births in England, 1986–1999	Office of National statistics birth recodes	Logistic regression	Morgan et al. 2004 [50]
	Dumpsite	Retrospective cohort study	All live singleton live births without congenital anomalies	Alaska Bureau of Vital statistics	Logistic regression	Gilbreath et al. 2006 [29]
	Industry	Case–control study	All births from the 1988 National Maternal and Infant Health Survey conducted in 48 states	National Maternal and Infant Health Survey (NMIHS)	Univariate and multivariate analyses	Sosniak et al. 1994 [30]
	Industry	Ecological study	All births in Teesside and Sunderland, 1986–1993	Office of population and censuses and survey	Unclear	Bhopal et al. 1999 [48]
			All singleton live births registred between 2004–2008	National institute for statistics	A Besag, York, and Mollié model	Castello et al. 2013 [53]
	HWS	Ecologic study	All births during 1994–2000 (excluded plural birth)	Birth certificate-based study	Multiple logistic models	Baibergenova et al. 2003 [28]
		Cohort study	All Live births and stillbirths, 1988–1998	Nova scotia atlee perinatal database	Logistic regression models	Dodds et al. 2001 [55]
	Incinerator	Retrospective cohort study	All births and fetal deaths in Japan, 1997–1998	Vital statistic records and birth certificate data	Stone’s unconditional test	Tango et al. 2004 [56]
LBW	Several site	Case–control study	All live births 1983–1985	Vital statistics files	Linear regression	Shaw et al. 1992 [23]
PTB	Landfill	Birth certificate-based study	All births	Birth certificate	Logistic regression model	Berry et al. 1997 [27]
		Case–control study	All live births to residents on Island of Montreal, 1979–1989 (excluded multiple birth and births to parous mothers)	Birth registration	Unconditional logistic regression	Goldbrg et al. 1995 [54]
	Waste	Cohort study	Live births and stillbirths, 1988–1998	Nova scotia atlee perinatal database	Logistic regression models	Dodds et al. 2001 [55]
	Dumpsite	Retrospective cohort study	All live singleton live births in Alaska Native villages without congenital anomalies	Birth records from the Alaska Bureau of Vital statistics	Logistic regression	Gilbreath et al. 2006 [29]
	Indurties	Ecologic study	All singleton live births registred between 2004–2008	National institute for statistics	A Besag, York, and Mollié model	Castello et al. 2013 [53]
SGA	Indurties	Ecologic study	All singleton live births registred between 2004–2008	National institute for statistics	A Besag, York, and Mollié model	Castello et al. 2013 [53]
	Landfill	Case–control study	All live births to residents on Island of Montreal, 1979–1989 (excluded multiple birth and births to parous mothers)	Birth registration	Unconditional logistic regression	Goldbrg et al. 1995 [54]
IURG	Waste site	Cohort study	Live births and stillbirths, 1988–1998	Nova scotia atlee perinatal database	Logistic regression models	Dodds et al. 2001 [55]
	Dumpsite	Retrospective cohort study	All live singleton live births in Alaska Native villages without congenital anomalies	Birth records from the Alaska Bureau of Vital statistics	Logistic regression	Gilbreath et al. 2006 [29]
Fetal /neonataldealth						
Stillbirth	Incinerators	Retrospective cohort study	All live birth and stillbirth	Birth certificate (Cumbriam birth database)	Multivariate logistic regression	Dummer et al. 2003c [44]
	Industrie	Retrospective cohort study	All live birth and stillbirth	Cohort-based study	Multivariate logistic regression	Dummer et al. 2003b [43]
		Ecological study	All births and stillbirths in Teesside and Sunderland, 1986–1993	Office of population and censuses and survey	Unclear	Bhopal et al. 1999 [48]
	Landfill	Ecologic study	Study of live births andstillbirths	National birth and stillbirth data	Poisson regression model	Elliott et al. 2001 [45]
			All births and stillbirths in Scotland between 1982 and 1997	National register-based study	Poisson regression model	Morris et al. 2003 [47]
		Retrospective cohort study	All live births and stillbirths	Cohort-based study	Multivariate logistic regression	Dummer et al. 2003a [42]
Neonatal death	Incinerators	Retrospective cohort study	All live birth and stillbirth	Birth certificate (Cumbriam birth database)	Multivariate logistic regression	Dummer et al. 2003c [44]
		Retrospective cohort study	All births and fetal deaths in Japan, 1997–1998	Vital statistic records and birth certificate data	Unconditional test	Tango et al. 2004 [56]
	Industrie	Retrospective cohort study	All live birth and stillbirth	Cohort-based study	Multivariate logistic regression	Dummer et al. 2003b [43]
	Landfill	Retrospective cohort study	All live birth and stillbirth	Cohort-based study	Multivariate logistic regression	Dummer et al. 2003a [42]
	Dumpsite	Retrospective cohort	All live births and fetal deaths in Alaska Native villages, 1997–2001	Birth records from the Alaska Bureau of Vital statistics	Logistic regression	Gilbreath and Kass 2006 [36]
	Crematorium	Retrospective cohort study	All live birth and stillbirth	Birth certificate (Cumbriam birth database)	Multivariate logistic regression	Dummer et al. 2003c [44]
Infant death	Incinerators	Retrospective cohort study	All births and fetal deaths in Japan, 1997–1998	Vital statistic records and birth certificate data	Unconditional test	Tango et al. 2004 [56]
	Waste site	Case–control study	All births	National maternal and infant health survey	Univariate and multivariate analyses	Sosniak et al. 1994 [30]
Fetal death	Waste site	Case–control	All births and fetal death	Birth and death records (Washington State vital records)	Multivariable logistic regression	Mueller et al. 2007 [40]
	Dumpsite	Retrospective cohort	All live births and fetal deaths in Alaska Native villages, 1997–2001	Birth records from the Alaska Bureau of Vital statistics	Logistic regression	Gilbreath et al. 2006 [36]
*All congenital anomaly combined*						
	Landfill	Ecologic study	Live births, stillbirths, congenital malformations including termination	Congenital register-based study	Poisson regression model	Elliott et al. 2001 [45]
		Ecologic study	All births, stillbirths, and termination registries in Scotland between 1982 and 1997	National register-based study	Poisson regression model	Morris et al. 2003 [47]
			Live and still born babies	Register of the office for national statistics	Poisson regression	Field et al. 2000 [21]
			Live births, stillbirths and termination	National congenital anomaly register-based study	Bayesian hierarchical logistic regression	Elliott et al. 2009 [11]
			Live births, late foetal deaths and terminations	The National Down’s Syndrome Cytogenetics Register (NDSCR)	Regression modelling within a bayesian framework	Jarup et al. 2007 [49]
		Cohort study	All birth (live birth)	UK office national	Logistic regression model	Palmer et al. 2005 [46]
		Retrospective cohort study	Live births and stillbirths	Cohort	Multivariate logistic regression	Dummer et al. 2003a [42]
		Case–control study	All live births, stillbirth, fetal deaths, and pregnancy terminations	Malformation register	Logistic regression models	Vriljheld et al. 2002a [51]
		Case–control study	Live births, stillbirths, and fetal deaths from 20 weeks gestation, and termination of pregnancy	Malformation register	Logistic regression models	Vriljheld et al. 2002b [1]
		Case–control study	All live births, stillbirth, fetal deaths, and pregnancy terminations	Malformation register	Logistic and related binomial regression models	Dolk et al. 1998 [52]
		Cohort and case–control studies		Regional congenital anomalies registry		Boyle et al. 2004 [41]
		Ecological study	All births	Danish Birth Defect Register	Risk rate calculated by dividing the sum of congenital anomalies by total birth	Kloppenborg et al. 2005 [26]
	Waste site	Case–control study	All live births, 1983–1984	Congenital malformations registry	Unconditional linear logistic regression	Geschwind et al. 1992 [37]
		Case–control study	All births and fetal deaths, 1983–1988;	Birth and fetal death certificate	Logistic regression model	Orr et al. 2002 [12]
		Case–control study	Study of births from the 1988 National Maternal and Infant Health Survey conducted in 48 states	National Maternal and Infant Health Survey	Univariate and multivariate analyses	Sosniak et al. 1994 [30]
		Case–control study	All singleton birth	The Newe York State Congenital Malformation Registry	Unconditional logistic regression	Marshall et al. 1997 [38]
			Singleton infant born alive or stillborn	California birth Defects Monitoring Program	Unconditional logistic regression	Croen et al. 1997 [35]
			All liveborn	Registry of all cases of confirmed heart disease born in Dallas County, Texas	Chi-square and mantel Haenzel analysis	Malik et al. 2004 [31]
			All live birth	Childrens’s Hospital of Wisconsin (CHW) and birth record	Logistic regression	Yauck et al. 2004 [22]
			Live births and fetal deaths	Texas Birth Defects Registry	Logistic regression	Brender et al. 2008 [33]
			Live births and fetal deaths (unless the termination had a vital record)	Texas Birth Defects Registry	Logistic regression	Brender et al. 2006 [32]
			Live births and fetal deaths of 20 weeks or greater gestation	Texas Birth Defects Registry	Logistic regression	Suarez et al. 2007 [39]
			Live births and fetal deaths	Texas Birth Defects Registry and birth or fetal death certificat	Logistic regression	Langlois et al. 2009 [13]
			Fetal deaths of ≥ 20 weeks gestation and live births,	Washington State vital record	Mantel haenzel analysis, logistic regression	Mueller et al. 2007 [40]
		Ecologic study	All live birth, stillbirths induced abortions and fetal death	Northern region Congenital Abnormality Survey (NorCAS)	Poisson regressions	Cresswel et al. 2003 [24]
		Cohort study	Live births and stillbirths, 1988–1998	Nova scotia atlee perinatal database	Logistic regression models	Dodds et al. 2001 [55]
		Descriptive geographical study	All birth defect cases and births during 1982–1989	Glasgow Register of Congenital anomalies	Poisson regressions	Eizaguirre et al. 2000 [25]
		Case–control study	All singleton infant born during 1987–2001	Birth-hospital discharge records	Multivariable logistic regression	Kuhen et al. 2007 [34]
	Industries	Ecological study	All births, stillbirths, and terminations in Teesside and Sunderland, 1986–1993	Congenital abnormalities register	Unclear	Bhopal et al. 1999 [48]
		Retrospective cohort study	All live births and stillbirths	Cohort-based study	Multivariate logistic regression	Dummer et al. 2003b [43]
		Ecological study	All live births and stillbirths	Regional medical center	Calculation of rate	Bentov et al. 2006 [57]
		Case–control study	All singleton birth	The Newe York State Congenital Malformation Registry	Unconditional logistic regression	Marshall et al. 1997 [38]
			All live birth	Childrens’s Hospital of Wisconsin (CHW) and birth record	Logistic regression	Yauck et al. 2004 [22]
			Live births and fetal deaths	Texas Birth Defects Registry	Logistic regression	Brender et al. 2008 [33]
		Case–control study	Live births and fetal deaths	Texas Birth Defects Registry	Logistic regression	Brender et al. 2008 [33]
			Live births and fetal deaths of 20 weeks or greater gestation	Texas Birth Defects Registry	Logistic regression	Suarez et al. 2007 [39]
			Live births and fetal deaths	Texas Birth Defects Registry and birth or fetal death certificat	Logistic regression	Langlois et al. 2009 [13]
	Dumpsite	Retrospective cohort	Live births and fetal deaths in Alaska Native villages, 1997–2001	Birth records from the Alaska Bureau of Vital statistics	Poisson regression	Gilbreath et al. 2006 [36]
	Several sites	Case–control study	All live births and fetal deaths 1983–1985	California Births Defects Monitoring Program	Logistic regression	Shaw et al. 1992 [23]
	Incinerators	Retrospective cohort	All births and fetal deaths in Japan, 1997–1998	Vital statistic records and birth certificate data	Stone’s unconditional test	Tango et al. 2004 [56]
		Retrospective cohort study	Live births and stillbirths	Birth certificate (Cumbriam birth database)	Multivariate logistic regression	Dummer et al. 2003 [44]
	Crematoriums	Retrospective Cohort study	Live births and stillbirths	Birth certificate (Cumbriam birth database)	Multivariate logistic regression	Dummer et al. 2003 [44]

### Reproductive outcome

The relation between maternal residence near sources of potential environmental hazard and pregnancy outcome has been investigated for a variety of outcomes. The first category, in number, is congenital malformations, encompassing studies of all congenital abnormalities combined [1, 11–13, 21–26, 30–39, 41, 45–48, 50, 52, 55, 57], specific abnormalities such as heart defects [1, 11–13, 22, 23, 26, 31, 34–36, 45–47, 52, 55], neural tube defects (NTD) [1, 11, 12, 35, 39, 45, 47, 52, 55], central nervous system abnormalities [12, 23, 26, 36–38, 52, 57], oral defects [11, 12, 23, 35, 37, 41, 45, 47, 52], chromosomal abnormalities [12, 13, 24, 33, 34, 37, 46, 49–51, 55, 57] and lethal congenital abnormalities [42–44, 56]. The second most investigated category of outcome encompassed low birth weight (LBW) [21, 23, 27–30, 45, 47, 48, 50, 53–56], preterm birth (PTB) [27, 29, 53–55], small for gestational age (SGA) [53, 54] and intrauterine growth retardation (IUGR) [29, 55]. The third outcome category was death, including infant death [30, 56], neonatal [36, 42–44, 56] or fetal death [30, 36, 40, 56], stillbirth [42–45, 47, 48] and spontaneous abortion [21].

### Exposure assessment

#### Sources of pollution

Most frequently, the pollution sources were hazardous waste sites [12, 13, 22, 24, 25, 28, 30–35, 37–40, 55] or landfills [1, 11, 21, 26, 27, 41, 42, 45–47, 49–52, 54]. Fewer papers have examined residential proximity to industries [13, 22, 32, 33, 38, 39, 43, 48, 53, 57], municipal waste incinerators [44, 56], dumpsites [29, 36] or crematoriums [44]. One study encompassed environmental risks from across landfills, dumpsites, hazard waste sites and industrial sites [23] (see Table 4).

**Table 4:** Summary of GIS-based (geographic information system) approaches used to assessed residential proximity to polluted site

Approach	Polluted sites	Study design	Exposure threshold	Study location	Auteurs, year
Distance-decay modeling	TRI	Case–control	0.5 mile	Texas	Suarez et al. 2007 [39]
			1.6 km (1 mile)	Texas	Langlois et al. 2009 [13]
				Texas	Brender et al. 2008 [33]
				Texas	Brender et al. 2006 [32]
				Texas	Suarez et al. 2007 [39]
			3.5 km (or 2 miles)	Texas	Suarez et al. 2007 [39]
			4.8 km (3 miles)	Texas	Suarez et al. 2007 [39]
		Cohort	Continuous measure	England	Dummer et al. 2003b [43]
		Ecological	3.5 km (or 2 miles)	Spain	Castello et al. 2013 [53]
	Waste site	Case–control	1.6 km (1 mile)	California	Croen et al. 1997 [35]
				California and New York	Sosniak et al. 1994 [30]
				Texas	Suarez et al. 2007 [39]
				Texas	Malik et al. 2004 [31]
				Texas	Brender et al. 2008 [33]
				Texas	Brender et al. 2006 [32]
				Texas	Langlois et al. 2009 [13]
			8 km (5 miles)	Washington state	Mueller et al. 2007 [40]
				Washington state	Kuehn et al. 2007 [34]
			Pondered distance	New York	Geschwind et al. 1992 [37]
	Landfill	Case–control	Continuous measure	5 pays européens	Vriljheld et al. 2002a [51]
			2 km	Wales	Palmer et al. 2005 [46]
		Cohort	Continuous measure	England	Dummer et al. 2003c [42]
	Incinerator	Cohort	Continuous measure	England	Dummer et al. 2003a [44]
	Crematoriums	Cohort	Continuous measure	England	Dummer et al. 2003 [44]
Buffer-based approach	Waste site	Case–control	1.6 km (1 mile, 1.32)	New York State	Marshall et al. 1997 [38]
				Milwaukee, Wisconsin	Yauck et al. 2004 [22]
		Ecological	3 km	New Castle upon Tyne	Cresswell et al. 2003 [24]
			A10 km subdivided into one circle of 2 km and1 km	Glasgow and nearby areas	Eizaguirre-García et al. 2000 [25]
	Landfill	Ecological	2 km	Great-britain	Elliott et al. 2001 [45]
				Scotland	Morris et al. 2003 [47]
				England and Wales	Jarup et al. 2007 [49]
				Denmark	Kloppenborg et al. 2005 [26]
			Exposure index-2 km	Great-britain	Elliott et al. 2009 [11]
			3 km	South Wales	Fielder et al. 2000 [21]
		Case–control	3 km	5 pays européens	Vriljheld et al. 2002a [51]
				5 pays européens	Vriljheld et al. 2002b [1]
				Europe	Dolk et al. 1998 [52]
			2–3 versus 4–5 km	Dublin, kildene, Wicklow	Boyle et al. 2004 [41]
		Cohort	3 km	England	Morgan et al. 2004 [50]
	Industry	Ecological	20 km	Beer-Sheva subdistrict	Bentov et al. 2006 [57]
	Incinerator	Cohort	2 km	Japan	Tango et al. 2004 [56]
Neighbor-based approach	Landfill	Ecological	NR	Philadelphia	Berry et al. 1997 [27]
		Case–control	NR	Montreal	Goldberg et al. 1995 [54]
	Industry	Ecological	NR	United Kingdom	Bhopal et al. 1999 [48]
Spatial coincidence	Waste site	Ecological	Zip-code	New York State	Baibergenova et al. 2003 [28]
		Case–control	Census tracts	California	Orr et al. 2002 [12]
				California	Croen et al. 1997 [35]
				San Francisco Bay Area	Shaw et al. 1992 [23]
		Cohort	City	Sydney, Nova Scotia	Dodds et al. 2001 [55]
	Dumpsites	Cohort	Villages	Alaska	Gilbreath et al. 2005a, b [29, 36]

*TRI* Toxic Release Inventory facilities

#### Exposure classification

Most studies have either considered sites generically (irrespective of their characteristics or the categories of pollutants emitted), or taken into account their specific characteristics.

#### Landfill sites

One study considered all landfill sites located within the study area [41]. Three of the papers investigated a single landfill site [21, 27, 54]. European studies based on the EUROHAZCON method selected sites that contained hazardous waste of non-domestic origin, as defined in the EC directive on hazardous waste [1, 50–52]. Palmer et al. [46], explored landfills that were licensed for storage of chemical waste and those that subsequently introduced containment and/or gas venting. In the same year, in Denmark, Klopen et al. [26] focused only on deposit and regular landfills which might have contaminated water and/or air, and which had been operating for more than 7 years prior to the start of their study.

Three studies [11, 45, 49] used the UK practice of co-disposal of special and non-special waste, and classified the waste by type that was handled and whether sites were licensed to store special hazardous waste (special/non-special, unknown). Special landfill sites are designed for co-disposal of hazardous, biodegradable and inert waste, whereas non-special landfill sites are designed for biodegradable and inert waste only (non-hazardous). On the basis of this classification, in 2003 Morris et al. studied the reproductive impact of residential proximity to special waste only [47]. Other studies used a more specific classification. Based on site files and Environment Agency classifications, Dummer et al. [42], assigned a code to each site that described the waste types treated, in order to rank them from lowest to highest potential toxicity: Type 1: inert, Type 2: nonhazardous, Type 3: household/putrescible, Type 4: difficult-to-handle [42]. Vrijheid et al. [1] used an expert panel scoring guide to obtain the hazard potential of a landfill site.

#### Hazardous waste sites (HWS)

Most studies relating to HWS have considered all categories of sites—with the exception of three, which explored: the reproductive impact of proximity to specific waste sites such as waste sites contaminated by polychlorinated biphenyl (PCB) [28] or those emitting TCE (trichloroethylene) [22]; one area polluted by chromium [25] and the Byker waste combustion plant [24].

Several studies exploring HWS employed the dedicated US-Environmental Protection Agency classification. Using the National Priority List (NPL) sites, including: inactive pesticide and chemical manufacturing plants, wood treatment and preserving facilities, drum storage facilities, mines, contaminated groundwater areas, sanitary landfills, and military bases), the authors studied any polluted site versus NPL-sites, non NPL-sites [13, 32, 33, 39] or all HWS versus HWS placed on a Superfund list (deemed higher-risk) [31]. Moreover, based on data characterizing the release of hazardous substances, the authors classified each hazardous waste site (both NPL and state Superfund sites) according to the environmental media contaminated and the specific chemical contaminants present—whether soil, surface water or air—were reported to be contaminated with heavy metals, PAHs or solvents [13, 32, 33, 35, 38, 39]. Also based on this US classification, Two other studies investigated residential proximity to NPL-sites [12, 30] and categorized NPL sites by those hazardous substances most present [12].

Another HWS classification was used by Mueller et al. [40] and Kuehn et al. [34] based on the hazardous potency of each site. Using the Washington Ranking Model (WARM), each site was rated on a scale ranging from of 1 (‘high-priority’ waste site) to 5 (‘low-priority’ waste site). Mueller et al. [40] also classified according to type of hazardous substance (solvents, metals, pesticides, radioactive substances) and contaminated media (water, drinking water, soil and sediment, air).

#### Industrial sites

Studies concerning industrial sites considered either any, or specific, industrial sites. Only two papers investigated a specific petrochemical industry [48], TCE emitting industry [22], or regional industrial park compound of 17 facilities [57]. Five US studies explored the health effects of all facilities taken together regardless of characteristics, or facilities classified according to their air releases, using information from the EPA Toxic Release Inventory. More precisely, the authors classified each industry by sector (petroleum refinery, primary metals or chemical industry) and by whether heavy metals or solvents were released [13, 32, 33, 38, 39]. Conversely, given that no specific data on emissions from hazardous industrial facilities is available in England, Dummer et al. [43] included all industrial sites that handled hazardous materials and chemicals, defined according to the Environment Agency register [43]. Lastly, in a recent European study, Castelló et al. [53]. investigated several types of industries—both as a whole and separately [53].

#### Incinerators

For this source, although the British study explored proximity to all incinerators [44], the Japanese one investigated only those incinerators having dioxin emission levels of above 80 ng TEQ/m^3^ [56].

### Confounding

Most studies adjusted for parental characteristics (e.g. maternal age, education, and marital status), birth characteristics (e.g. parity, number of previous stillbirths, gender of baby, gravidity, prior fetal death, etc.) and unhealthy practices (maternal smoking and alcohol consumption during pregnancy). Because of the lack of available information on dietary factors (such as folic acid supplementation, folic acid and vitamin intake during pregnancy), few studies have adjusted risk estimates for these variables. Some authors did take account of other confounders; a few studies adjusted for other exposure sources, such as parental occupation with relevant exposures (e.g. exposure to solvents or metals), or neighborhood characteristics (census tract median income, population density, urban versus rural residence, neighborhood socio-economic status).

## Methodology for spatial definition of residential proximity to polluted sites

The choice of GIS approach differs between studies according to the type of polluted sites (HWS, landfill, or industrial site) and the study design which was conducted.

### GIS-based approach

We identified four main GIS-based approaches used to delineate population at risk close to polluted sites: (i) the approach based on distance-decay modeling, (ii) the buffer-based approach, (iii) the spatial coincidence method and (vi) the neighbor-based approach (see Table 4).

#### Potential exposure of a population living close to hazardous industrial facilities

The main method used to estimate the potential exposure of a population living close to hazardous industrial facilities was based on distance-decay modeling method [13, 32, 33, 39, 43, 53] with the exception of one study based that used buffer method [57] and another neighbhorhood-based approach [48].

The authors used GIS tools to measure the straight-line distance between the location of the study population and the nearest polluted site. Depending on data availability, the location of the population was based either on individual data (mother’s residence [13, 32, 33, 39]) or on data aggregated across geographic units such as postcodes [53]. Some studies have extended this general concept to compute individual proximity measures. For example, Dummer et al. [43] conducted an individual study whereby for each birth, an individual proximity measure, λ, was calculated using the following formula,

$$ \lambda = \frac{1}{{\left( {{\text{D}} + 0. 1} \right)^{2} }} $$

where D is the distance from polluted site to mother’s residence. The measure was summed over all sites in operation, covering the study territory at birthdate.

More recently, Castelo et al. [53]. estimated maternal exposure to industrial pollution by taking into account the distance from the administrative center of the residential municipality to the pollution source, using a purpose-designed distance matrix between all industrial installations and all municipalities.

Moreover, to investigate proximity to industrial site, Bentov et al. constructed a buffer to delineate the zone at risk for each industrial sites [57], while Bhopal et al. [48], aggregated several neighborhoods into 3 zones (A, B, and C—with A being closest to industry and C most distant). They did not, however, explain their criteria used to define each zone.

#### Potential exposure of a population living close to landfill sites

The main method used to estimate the potential exposure of a population living close to landfill sites was based on buffer method [1, 11, 21, 27, 41, 45, 47, 49–52] with the exception of three studies based on Distance-Decay Modeling Method [42, 46, 51] and two others based on neighborhood-based approach [27, 54].

For instance, to investigate proximity to a landfill site, a circle of predefined radius is drawn around the polluted site. Some studies have extended this simple concept to calculate a landfill exposure index. Elliot et al. [11] addressed the issue of multiple exposures, exploring variation in risk of congenital abnormalities among areas hosting differing geographic landfill site densities by calculating an index based on the number of hazard zones (using a 2 km radius circle around each landfill site) overlapping each postcode. The resulting number was then related to birth data, and aggregated over a 5 × 5 km grid [11].

In some European studies based on the EUROHAZCON approach, and in one US study, an area of 7 km radius around each landfill defined a ‘study area’. Each study area contained a ‘proximate’ zone of 3 km radius from the site, within which the population was considered to be most exposed to chemical contaminants. This was compared to the ‘unexposed’ population within ‘distal’ zones of 3–7 km [1, 47, 50–52].

In 1995, Goldberg et al. [54] defined a set of three putative exposure zones representing proximal and distal areas to a municipal solid waste landfill site. These zones were formed by grouping contiguous or near-contiguous postal code areas. The high exposure zone consisted of the postal code area in which the waste landfill site was located, or which bordered it. In another US study conducted in 1999, Berry et al. defined exposed mothers as those living closest to the Lipari landfill in the only neighborhood next to the landfill [27].

#### Potential exposure of a population living close to hazard waste site

The main method used to estimate the potential exposure of a population living close to hazardous HWS was based on Distance-Decay Modeling Method [13, 30–35, 37, 39, 40] followed by five others studies based on buffer method [22, 24–26, 38] and five others based on spatial coincidence method [12, 23, 28, 35, 55].

To measure the proximity to HWS using the straight-line distance, the location of the population was based either on individual data (mother’s residence [13, 31–35, 37, 39, 40]) or on data aggregated across geographic units such as zip code centroids or postcodes [30].

In 1992, Geschwind et al. created an individual ‘exposure risk index’ incorporating distance from, and the hazard raking score, for each site within a 1-mile radius of birth residence [37]. Thus, the higher the waste site score and the closer an individual’s proximity to a site, the greater the weighting factor assigned.

Some authors entails constructing a buffer to delineate the zone and population at risk for each HWS (see above in “Potential exposure of a population living close to hazardous industrial facilities” section) to compare to the ‘exposed’ population in ‘proximate’ zone with ‘unexposed’ in ‘distal’ zones [22, 24, 25, 38].

While, Croen et al. [35] defined a measure of proximity as one indicating whether the census tract of residence contained a waste site. Baibergenova et al. [28] defined exposed groups as those residing in a zip code hosting PCB-contaminated sites, and unexposed groups as residing in zip codes not hosting PCB-contaminated sites. Orr et al. [12] considered that where a census tract contained one or more NPL sites, the children born in that census tract were considered to be ‘potentially exposed’. These studies used a variety of spatial units with different resolution scale (zip code, census block) [12, 28, 35].

#### Potential exposure of a population living close to specific hazard waste site

Among studies focusing on excess risk of pregnancy outcome associated with living near specific HWS, different method used to estimate the potential exposure of a population living close to specific HWS (including incinerator [44, 56], dumpsite [29, 36] and crematoriums [44]) was based on Distance-Decay Modeling Method [44], buffer method [56] and spatial coincidence [29, 36].

Some studies have extended a general concept to examine the relationship between reproductive outcome and nuisance intensity, measured by the amount of chemical released or by the toxic potency of the chemicals. For example, several authors [29, 36] investigated whether women living in villages having ‘highly hazardous’ open dumpsites had greater rates of adverse pregnancy outcome than women living in villages with sites having a lower hazard ranking.

### GIS-based approach according the study design

Except one ecological study [53], all studies which used distance-decay modeling method to estimate the potential exposure of a population living close to polluted site were individual studies including mainly case–control [13, 30–35, 37, 39, 40, 51] and also four cohort studies [42–44, 46].

Most ecological studies including one descriptive geographic study [11, 21, 24–26, 45, 47, 49, 57] and several case–control studies [1, 22, 38, 41, 51, 52] used buffer method to investigate the excess risk of pregnancy outcome associated with living near polluted sites, while only three cohort studies [50, 56] used this method to estimate the potential exposure of a population living close to polluted sites.

Similarly, while no cohort study used neighborhood based approach, two ecological [27, 48] and one case–control study [54] used this approach to estimate the potential exposure of a population living close to polluted sites.

In contrast, among few studies which used spatial coincidence method, the most studies were individual including three cohort studies [29, 36, 55] and three case–control studies [12, 23, 35], and only one ecological study [28] used this approach to estimate the potential exposure of a population living close to polluted sites.

### Exposure threshold

The definition of residential exposure zone differs between studies according to the type of polluted sites (HWS, landfill, or industrial site) and the country in which the study was conducted.

In most US studies, exposed women were located within either 1 mile of waste sites [13, 22, 23, 30–33, 35, 39] or 5 miles [34, 40], though distances of 3 km [24] or 2 km [25] from waste sites or specific HWS were also found. In a few European studies and one other US study, exposed women were those who lived within either 3 miles [38, 53] or 1 mile of industrial sites [13, 32, 33, 39]. Most analyses based on buffer methods defined areas of 1 km [27], 2 km [11, 26, 45, 47, 49] or 3 km [1, 21, 51, 52] radius around each landfill site as being ‘zone-proximate’ and thus classified as ‘exposed’.

## Overview of current evidence concerning possible effects on pregnancy outcome of proximity to polluted sites

In this section, the results of studies are structured by type of polluted site, namely (i) industrial site (ii) HWS, (iii) Landfill site and (vi) incinerator/dumpsite.

### Risk of adverse pregnancy outcome around industrial site: (Fig. 2)

Among studies focusing on excess risk of pregnancy outcome associated with living near industrial facilities, results show that the risk of PTB or very PTB [53] stillbirth [43, 48] and neonatal death [43] were not found to be associated with living in close proximity to specific industries [43, 48]. However, other studies show an increase in the prevalence of LBW and MLBW, as well as risk of SGA, with residential proximity to industrial facilities from different sectors of activity (RR = 1.03; 1.01–1.05 [53]; OR = 1.13 (0.99–1.30) [48]; RR = 1.03; 1.01–1.05 [53] respectively).

**Fig. 2:**
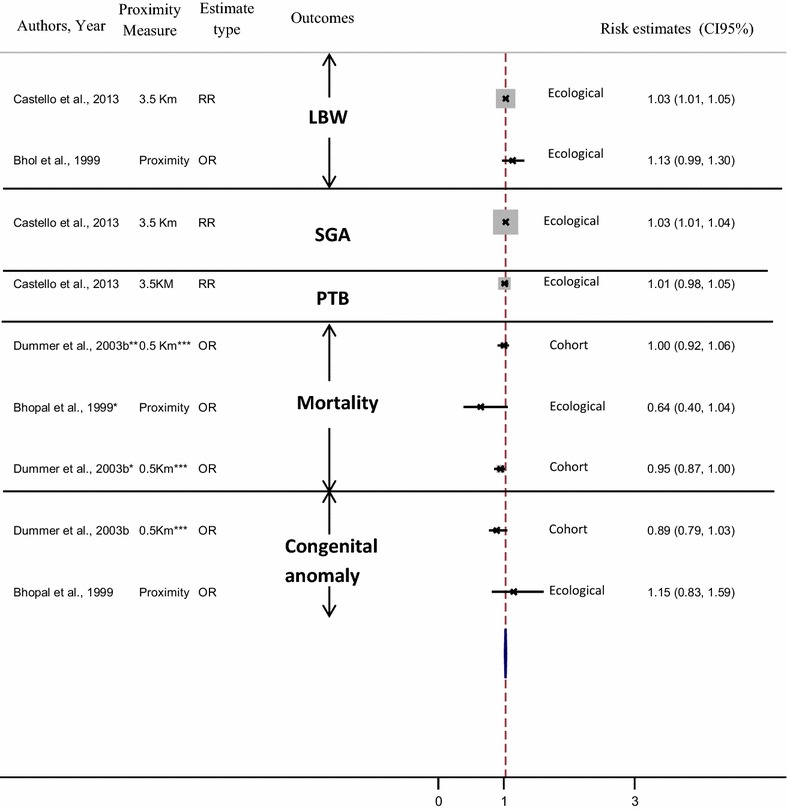
Risk of adverse pregnancy outcome around industrial site. (*LBW* low birth weight, *PTB* preterm birth, *SGA* small for gestational age). *Stillbirth; **neonatal mortality. ***OR comparing odds at a distance of 0.5 km with that at a distance of 10 km

In addition, with the exception of two studies which reported no association between proximity to industrial sites and all congenital anomalies combined [43, 48], our review reveals that women living close to industrial sites have an increased risk of giving birth to children with:

- overall congenital malformations (RR = 1.17; 1.04–1.29-among Bedouin populations—[57]),
- chromosomal abnormalities (OR = 4.8; 1.2–42.8 only among women aged 40+ [33]),
- specific congenital malformations including neural tube defects (OR = 1.2; 1.0, 1.5 [39]) and Congenital Heart defects (CHD) (OR = 3.2; 1.2–8.7 [22] with proximity to trichloroethylene-emitting sites,
- increased risk of death from congenital heart defects (OR = 1.06; 1.02–1.10 in 1983–1993 [43].

### Risk of adverse pregnancy outcome around landfill sites: (Fig. 3)

Among studies focusing on the relationship between pregnancy outcome risk and residence near landfill, the results reveal that the risk of mortality including: stillbirth [42, 45, 47], spontaneous abortion [21], neonatal death [42] and SGA [54] was not found to be associated with living in close proximity to landfill [45, 54] or specific landfill [21, 42, 45, 47].

**Fig. 3:**
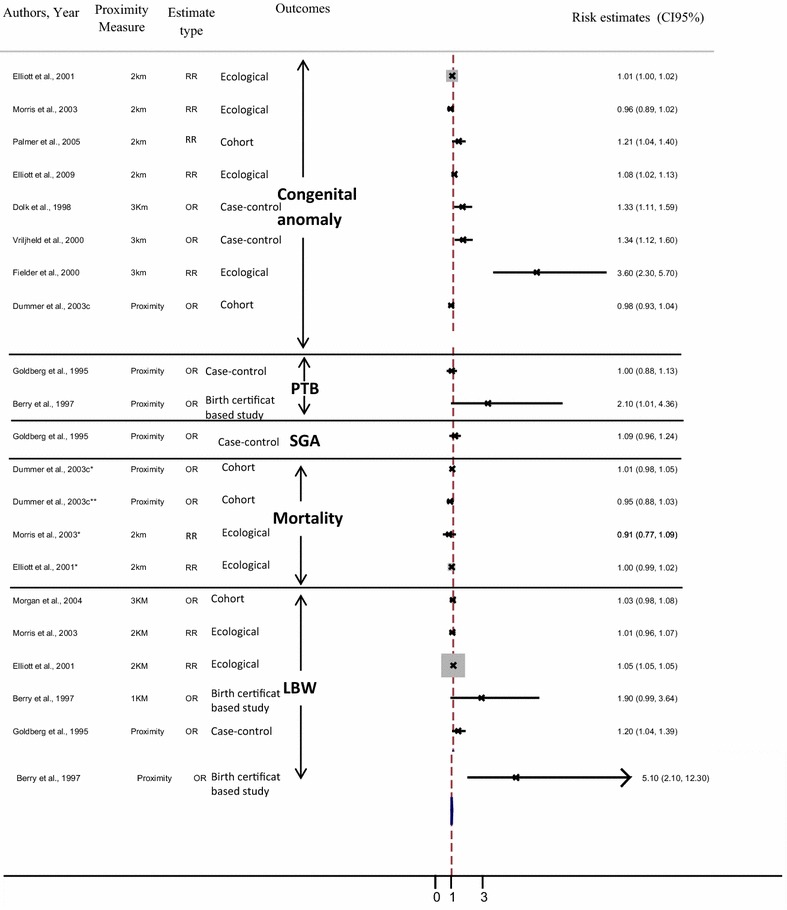
Risk of adverse pregnancy outcome around landfill sites. (*LBW* low birth weight, *PTB* preterm birth, *SGA* small for gestational age). *Stillbirth; **neonatal mortality

However, an increase in the prevalence of very LBW [45], LBW and risk of PTB with residential proximity to landfills (RR = 1.04; 1.03–1.05) [45]; RR = 1.05; 1.05–1.06 [45]; OR = 5.1; 2.1–12.3 [27]; OR = 1.20; 1.20–1.39 [54]; OR = 2.10; 1.01–4.36) [27] respectively) were revealed by three studies—even though three other studies indicate that no statistically significant excess risks of LBW and PTB or very PTB [54] were detected in populations living near landfill [21, 47, 50, 54].

In addition, an increased risk of congenital abnormalities was found in the children of mothers living near:

- waste landfill (RR = 1.01; 1.01–1.02 [45]; OR = 1.33; 1.11–1.59 [1]; RR = 1.9; 1.3–2.85; before versus after opening RR = 1.9; 1.23–2.95 [21] and RR = 3.6; 2.3–5.7 when site being developed and first used [21],
- special waste landfill (RR = 1.07; 1.04–1.09) [45]; OR = 1.08; 1.02–1.13 [11]),
- landfill with chemical waste (OR = 1.21; 1.04–1.40) [46] or waste of medium hazardous category (OR = 1.48; 1.19–1.85) [1], non domestic hazardous waste (OR = 1.33; 1.11–1.59) [52].
- non-domestic waste landfill (OR = 1.41; 1.00–1.99 for chromosomal abnormalities [51],

Moreover, studies reveal that women living close to a landfill site had:

- an increased risk of giving birth to children with specific congenital malformations including neural tube defects (OR = 1.86; 1.24–2.79 [52]; RR = 1.05; 1.01–1.10 [45]), cardiovascular defects OR = 1.16; 1.01–1.33 [11]); anomalies of great arteries and veins OR = 1.81; 1.02–3.20 [52] or cardiac septa OR = 1.49; 1.09–2.04) [52],
- increased risk of death from ‘other congenital abnormalities of the nervous system’ closer to domestic waste landfill sites (continuous OR = 1.14; 1.03–1.25 for increasing proximity to landfill sites [42]).

However, three studies reported that no statistically significant excess risks of congenital abnormalities were detected in populations living around landfill sites [26, 41, 47].

### Risk of adverse pregnancy outcome around hazard waste sites: (Fig. 4)

Among studies focusing on relation between maternal residential proximity to HWS and adverse pregnancy outcome, results show that the risk of PTB was significantly elevated among infants born to women living near HWS (RR = 1.13; 1.04–1.22) [55], but the risks of infant death [30], fetal death [40] and risks of low [30], very low birth weight [30] and IURG [55] were not found to be associated with living in close proximity to HWS [30, 40, 55] or NPL HWS [30].

**Fig. 4:**
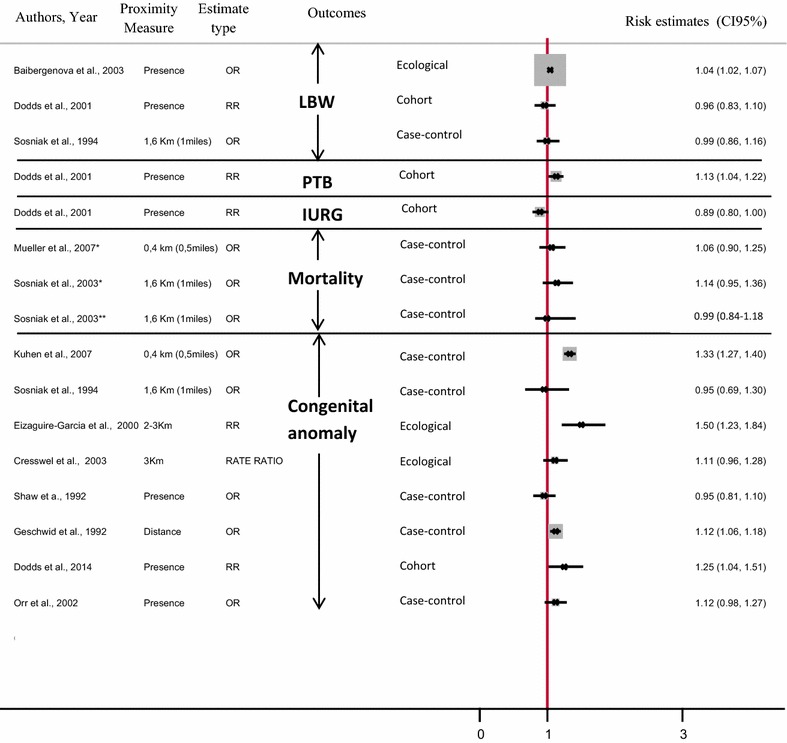
Risk of adverse pregnancy outcome around HWS. (*IUGR* intrauterine growth retardation, *PTB* preterm birth). *Fetal mortality; **infant death

Among studies focusing on specific sites, studies showed an excess risk of LBW with proximity to PCB-contaminated waste sites (OR = 1.04; 1.02–1.07) [28] and higher risk of fetal death among women residing close to pesticide-containing sites (OR = 1.28; 1.13–1.46) [40].

In addition, authors found a significant increase in the risk of congenital malformations among women living close to HWS (OR = 1.12; 1.06–1.18 [37]; OR = 1.15; 1.10–1.21 [34]; RR = 1.25; 1.04–1.51 [55]), with the exception of one; Sosniak et al. found that maternal residential proximity to NPL sites was not associated with adverse pregnancy outcome including: congenital abnormalities [30].

Moreover, authors revealed that an increased risk of congenital malformations was found only with proximity to specific waste sites including:

- waste sites emitting substances with specific biological effects (cytochrome oxidase inhibitors) (OR = 1.3; 1.02–1.67) [12],
- chromium waste (RR = 1.52; 1.24–1.85) [25],
- waste sites classified as ‘high priority’ (OR = 1.16; 1.11–1.20) [34].

Moreover, women living close to HWS had an increased risk of giving birth to children with specific congenital malformations including neural tube defects (RR = 1.83; 1.08–3.09 [55]), and cardiovascular defects (OR = 1.20; 1.1–1.4 [31]; OR = 4.99; 1.26, 14.51 [13]).

### Risk of adverse pregnancy outcome around specific waste sites: (Fig. 5)

Among the studies focusing on the relationship between maternal residential proximity to specific waste sites and adverse pregnancy outcome, results reveal that risks of mortality including: stillbirth [44], neonatal death [44, 56] and infant death [56], and risk of LBW [56], were not found to be associated with living in close proximity to polluted incinerators [44] and specific incinerators [56] and crematoriums [44]). However, Dummer et al. [44] described a higher risk of stillbirth among residents (OR = 1.04; 1.0.1–1.07).

**Fig. 5:**
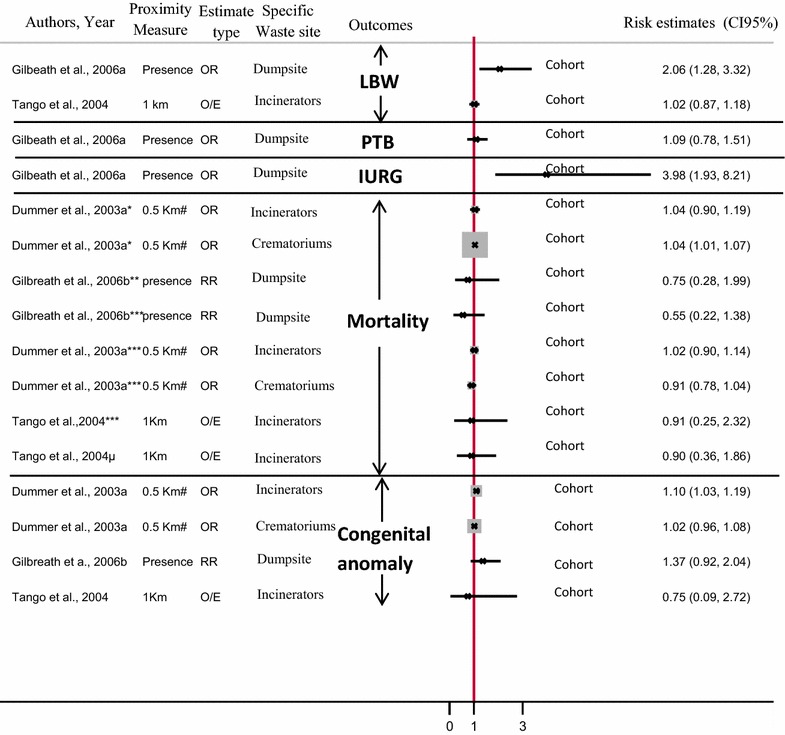
Risk of adverse pregnancy outcome around specific waste sites. (*LBW* low birth weight, *PTB* preterm birth, *IUGR* intrauterine growth retardation). *Stillbirth; **fetal death; ***neonatal mortality; ^µ^infant death. ^#^OR comparing odds at a distance of 0.5 km with that at a distance of 10 km

Dummer et al. also found a significant increase in the risk of lethal congenital malformations (OR = 1.10; 1.03–1.19) [44], lethal specific congenital abnormalities including neural tube defects (OR = 1.13; 1.04–1.23) [44] and heart defects (OR = 1.12; 1.03–1.22) [44] among women living close to incinerators but not around crematoriums [44].

Whereas in 2006 Gilbreath et al. revealed increased risk of IUGR and prevalence of LBW around the dumpsite (OR = 3.98; 1.93–8.21; OR = 2.06; 1.28–3.32) [29] respectively), these same authors have shown that risk of PTB [29] or very PTB [29], risk of neonatal death [36], fetal death [36] and congenital anomalies [36] were not found to be associated with living in close proximity to dumpsites [29] or specific dumpsites [36].

## Discussion

### Main findings

Based on cohort and case–control studies, our systematic review has shown the strength of the association between adverse pregnancy outcome and maternal residential proximity to polluted sites to be highly variable. Increased risks for non-chromosomal abnormalities, chromosomal abnormalities, low birth weight and small for gestational age were noted in several U.S. and European studies among populations living close to hazardous waste sites—yet measures of association were not significant for other types of birth defects.

Our review mainly reveals an excess risk of reproductive morbidity—though not of mortality. Despite several non-significant associations, Fig. 2 shows that all published studies are on the side of an increased risk of congenital abnormalities. In addition, Fig. 2 shows that all but four studies exhibited an excess risk of low birth weight. Results for preterm birth [27, 29, 53–55], SGA [53, 54] and IUGR [29, 55] convey the same pattern (see Figs. 2, 3, 4, 5).

Our literature review highlights the fact that the differing findings of studies may, in part, be due to how ‘proximity of residence’ is assessed. For example, in a study involving five European countries and 23 hazardous waste landfill sites, women who lived within 3 km (1.9 miles) of such a site were 1.5 times (95% CI 1.0–2.2) more at risk of chromosomal abnormalities than women living in the 3–7 km band [51]. On the other hand, in 2008 [33], Brender et al. found no association between living near hazardous waste sites and chromosomal abnormalities (OR = 0.90; 0.70–1.2). In a study among California residents of maternal residence near NPL waste sites and birth defects, women who lived in a census tract having one or more NPL sites were more likely to have births with congenital abnormalities (Patau syndrome or Edward’s syndrome or other sex chromosome abnormalities OR = 2.65; 1.37–5.13; OR = 2.7; 1.53–4.61; OR = 3.1; 1.01–9.62, respectively) [12].

These contrasted results could be partially explained by methodological limitations inherent to (i) exposure assessment, (ii) the GIS methods, which could also affect the strength of association. In addition, several inaccuracies and biases, inherent to different analysis methods, may bias cross-study comparisons and conclusions drawn from them. These limitations will be discussed below in the second part.

### Exposure assessment

The main limitation of the studies reviewed in the present paper lies in exposure assessment, which comprises *(i) categorization of exposure sources and (ii) factors influencing the potential exposure to polluted sites.*

#### Categorization of exposure sources

We sum up the four main methodological limitations regarding the categorization of exposure sources that may yield exposure misclassification.

Firstly, in some cases (such as practice of co-disposal in the UK) the two categories of special and non-special waste may not necessarily correspond to higher levels of hazard in the former, as has been hypothesized by some authors. The special waste sites may handle smaller volumes of hazardous waste and be subject to stricter management and design standards than other non-specialized waste sites, at which hazardous wastes may have been disposed of unreported.

Secondly, most US studies have included sites with ‘unknown waste’ in the analysis. The large number of such sites provides potential for uncertainty as to their degree of hazardousness. In practice, sites were likely to be of unknown type for three main reasons: (i) because they were legally not subject to regulation due to the nature of their operation (e.g., small dump sites in farms, taking agricultural waste from the holding), (ii) because they were not active during the study period, or (iii) because they were informal sites not identified by the competent authorities. Thirdly, with the exception of studies of special waste sites, most studies were based on polluted sites falling into more than one hazardous substances category—and in addition, some census tracts contained more than one site. In such instances, the association cannot be ascribed to a particular category of pollution or site [12].

Lastly, in situations where pollution remediation (or at least containment) may be in process, it is likely that exposures of neighboring residences have been reduced. This might explain those cases where no association was found between maternal residence and chromosomal abnormalities in offspring [32, 33].

#### Factors influencing potential exposure to polluted sites: consideration of dispersion factors

Residential exposure to site contaminants and industrial emissions also varies according to climatic and topographic characteristics such as prevailing wind speed, direction and temperature. Within the constraints of the available data—including lack of geological, meteorological, or water supply information—these conditions were rarely accommodated except for few studies [27, 54, 57]. Whatever the measure used by studies to estimate the potential exposure of a population living close to polluted sites including both buffer-based and distance-based approaches, the author not consider dispersion factors—the reduction of personal exposure to a simple distance function is restrictive. Most other studies ignored this point; they considered emissions from a facility to be uniformly dispersed in all directions, and environmental exposure to be equally distributed around the polluted sites. Yet a resident who lives one mile upwind of a hazardous facility is unlikely to experience the same level of exposure as someone living one mile downwind. According to the study by Brender et al. [33], this point may particularly impact women living close to two or more facilities.

### GIS-based methodology

Since use of GIS tools is now widespread, computing proximity-based indicators is fast, easy and applicable to large data sets. Basic GIS functions, such as point-in polygon, intersect, or buffering distance are used. Moreover, to assess polluted site exposure, GIS-based approaches seem pertinent to explore “*geophysical plausibility*”—a new term coined and described by Nuckols et al. [58]. To use in the application of environmental science to exposure assessment for epidemiology, they suggest this axiom which would dictate that: “an association between a contaminant source and exposure to an organism or ecologic community cannot exist unless there is a plausible geophysical route of transport for the contaminant between the source and the receptor” [58].

However, these proximity indicators may bias assessment of residential exposure due to GIS-approach procedures used to define proximity to polluted sites. Studies using spatial coincidence methods are limited by their inability to consider the exact geographic location of the hazard within the host spatial unit and determine the geographic extent of exposure. In order to address the limitations of the spatial coincidence approach, most studies have analyzed residential proximity either on the basis of distance, or using the buffer method. Buffer methods and distance based-approach analysis provide more accurate and realistic estimates of exposure than spatial coincidence methods because they do not assume that adverse effects are restricted to the boundaries of the pre-defined analytical units hosting the hazard source. However, there are specific limitations associated with its application, with various sources of both error and uncertainty, i.e. *(i) physical geography of the facility; (ii) definition of residential proximity: “Geophysical Plausibility”.*

#### Physical geography of the facility

Most studies have assumed that the facility or contamination site was small enough to be treated as a point source; few have considered their shape and size in deciding which type of buffer was appropriate [32, 33, 35]. Yet landfill sites vary greatly in terms of surface area, from 50 m^2^ to 70 million m^2^ (average 64,600 m^2^ in the study base) [49], and areas and locations do change over time as sites evolve. The use of a point location to define sites yields uncertainties. Some hazardous sites should be polygon-delineated, with the buffer should be constructed around this shape [59].

Moreover, the properties and quantities of hazardous substances stored or released at each facility have rarely been incorporated to the determination of buffer radii to reflect the spatial extent of environmental exposure. Nor are the operational parameters of emission releases (e.g. release height, exit velocity, exit temperature) considered in determining buffer size.

#### Definition of residential proximity: “geophysical plausibility”

Misclassification of exposure may also arise out of the variety of radii or distances used (1, 2 or 3 km) to define proximity to polluted sites. Our review highlights the fact that radii of the circular buffers and distances defining maternal exposure have been chosen arbitrarily. Few authors have justified their choices [1, 11, 13, 45, 47, 49, 51, 52]. The conclusion from a WHO report [60] had guided several authors who stated in their paper that exposure from landfill sites is likely to be limited beyond 1 km from the site by the air pathway, and 2 km by the water pathway [11, 45, 49]. Other authors based their choice on expert judgment, positing that exposure to chemical contaminants would occur within a 3 km radius of landfill sites [1, 51, 52]. In order to be consistent with most American studies of waste sites or industrial facilities in relation to birth defects, several authors chose to use the same radius [13, 47].

Irrespective of buffer size, there is some intrinsic inaccuracy in drawing such exposure areas [42]. In the absence of finer resolution information, and because of the complex nature of sites such as landfills, use of distance bands smaller than 2 km or a continuous measure to examine proxy dose–response relationships would have been beyond the resolution of the data [45, 49]. While arguing that it is not possible to detect directional patterns using concentric circles, Palmer et al. [46] supported the *idea* that the use of 2 km radii, as chosen by Elliott et al. [11, 45] was pragmatic, maximizing the power of comparisons while remaining within plausible estimates of the range of chemicals dispersed from a site. No sound evidence has yet been published to assess human exposure with distance from landfills in the United Kingdom, but expert opinion suggests that small particles from landfills may be detectable up to 3 km away [60]. Alternatives to using concentric circles could be explored, given that the distribution of increased risk is not uniform with distance [61].

However, as explained by Elliott et al., distance from the nearest landfill site may not be regarded as a meaningful proxy for exposure where postcodes have been used to define the location of birth outcomes and where point locations had to be used for estimation of polluted sites, particularly in rural areas [11, 45].

### Assessment of the relation between spatial proximity to polluted site and reproductive outcome

Interpretation of our findings must consider weaknesses that could affect the strength of the associations, yield limitations in comparisons or impede the formulation of accurate conclusions. These weaknesses, discussed below, are inherent to (i) outcome data, including the definition and the selection of the case, (ii) study design, and (iii) assessment of the risk of pregnancy outcome around polluted sites. In addition, beyond these factors, the systematic review we conducted also faced some methodological limitations.

#### Outcome data-case selection

There are several ways in which outcome data can be a source of bias. Firstly, findings may be distorted by selection bias. Examples given by some authors are exclusion of pregnancies terminated prior to 20 weeks of gestational age [12, 13, 39] and pregnancy terminations without vital records [33]. This tends to bias association estimates towards low values and might even—at the extreme—reverse the direction of the true association because those women less likely to terminate pregnancies in conjunction with less frequent usage of prenatal diagnosis, lack of access to safe delivery facilities (e.g., poor women), or cultural practices (e.g., Hispanic women) may also be more likely to live closer to industries and waste sites [13, 33, 39].

Similarly, the population source between studies differs, having a potential impact on association measures. Whereas some authors collected their data from population-based studies encompassing all live births, fetal deaths and other pregnancy terminations, others had information only on live births—thus restricting ascertainment of birth defects [34, 37, 38].

One source of such limitation lies in the databases. Using linked birth-hospital discharge data may reduce the likelihood of missing malformations, because it includes malformations identified throughout birth hospitalization, rather than only at birth. Malformations resulting in early fetal death or elected termination, if not included, may yield the same effect, so that risk estimates of CNS and chromosomal malformations, in particular, may be inaccurate.

Outcome definition is another source of uncertainty. Unlike low birth weight (weight <2500 g) and preterm birth (<37 week), the definition of congenital malformation was heterogeneous across studies, rending comparisons difficult. Some studies have excluded non-lethal congenital abnormalities [42–44], whereas some included only live births with congenital malformations [22, 26, 31, 34, 37, 38, 41, 46] and others included both live birth and fetal death with birth defect [12, 24, 32, 33, 39, 57]. Broad groupings of malformations into *all congenital abnormalities combined* may also have hampered the ability to examine associations for specific malformation types by diluting relevant cases [34]. The loss of precision inherent to such a general classification scheme (e.g. malformations placed within the same classification grouping) reduces the likelihood of detecting an association between malformations and the study exposures [37].

#### Study design

The ecological studies are all published from the 2000s as the cohort studies. While, in this work, a majority of the studies were conducted in US, we count 9 ecological studies realized in Europe, and only 2 in US [27, 28], and one in Israel [57]. Similarly, only one of the cohort study was conducted in US [36] whereas a majority came from UK. We count 19 case–controls studies conducted between 1992 and 2009. Inversely to ecological and cases–controls studies, a huge majority of the cases–controls studies were conducted in US; only four studies in Europe [1, 41, 51, 52] and one in Canada [54].

The outcomes most frequently investigated in the ecological is not the congenital abnormalities (as we observed when considering overall studies) but the birth outcome such as LBW, preterm birth, etc; (seven over the eleven ecological studies) whereas the inverse situation was observed in the cases–controls studies with a majority of studies dealing with the congenital abnormalities—only one study investigated the LBW and preterm birth outcome [54], and another one the fetal death [40]; two last one included various outcomes as LBW, fetal and infant death and congenital anomalies [23, 30]. The cohort study design is the only one for which, many studies dealt with death event: Infant, neonatal, and fetal deaths besides congenital malformations and ‘classical’ birth outcomes as LBW or preterm birth.

The landfill is the polluted site most frequently investigated in the ecological and cohort studies whereas in the cases–controls studies the polluted sites of interest were the hazardous waste and industrial sites. We also highlighted that the Europe cases–controls studies investigated landfill polluted sites which is coherent with the ecological and cohort studies.

The study designs could impact the quality of each study included in the review and consequently make difficult the comparison between studies. In addition, the study may impact *(i) the measure of women exposure and (ii) the risk estimate.*

##### Study design and the measure of women exposure

The different study designs present itself strengths and limitations to measure the proximity to polluted site according to the available data.

##### Ecological approach

In the ecological studies, the authors do not measure the exposure at the individual level, thus their results depends on the scale of spatial unit in which the indicator of exposure was estimate.

When the place of residence of each case is no known, the individual approach is no possible, and an ecological study is recommended. However, misclassifications of exposure may result from the use of municipality [53], or zip code [28] to define the location of maternal residence. Postcodes provide only an approximate location of the residential place. With an average of about 12 households per postcode in urban areas with high population density [11], it corresponds to a very small area. In contrast, in remote rural areas, a single postcode may cover an area of 1 km^2^ or more. Thus, there is the possibility for systematic bias in the exposure estimates (with less precise estimates in rural areas). Adjustment for rurality was partially controlled for this problem in the 2009 study by Elliott et al. [11].

Moreover, in ecological study, for which residential places are not known with precision, the indicator chosen to estimate exposure level is the mean which ignore the variability of exposure within the census block scale or zip code. Therefore, in the ecological approach, all women living within a given spatial unit have an equal exposure level, this presumption is known as ecological fallacy.

For instance, when authors used spatial coincidence methods to measure of women exposure based on the presence of polluted sites within a particular spatial unit of aggregation, the authors suggest that all women living within a particular boundary are all impacted equally by the hazard of interest, without an accurate assessment of individual exposure.

Whereas, women living next to polluted site but this hazard is not located within their spatial unit would not be defined as exposed. Therefore, the larger the spatial unit, the more likely it is that bias will be introduced due to heterogeneity within these units, and ecological fallacy may result.

However, when precise information concerning the individual location is missing ecological studies constitute an appropriate alternative to investigate some hypotheses. These approaches are easy to perform in a short period of time, and at a low cost. In addition, they are less likely to show random variation errors than analytic case–control studies.

##### Individual approach

When the place of the residence of each women is well known, the individual approach is possible. However, incorrect geocoding of both the residential addresses of the pregnant women and the polluted sites may bias the study’s findings. Firstly, the process of geocoding may itself introduce bias because geocoding accuracy depends on many characteristics. For instance, accuracy is higher in urban than in rural areas, because rural addresses are frequently reduce to the name of a street (with no number) or to the place name (with no street/road name) [62]. The result of the geocoding process may bias the study population as several studies revealed that subjects whose addresses were not geocoded tended to be younger, Hispanic and less well-educated than subjects whose addresses were geocoded [39]. However, most case–control studies indicated that un-geocoded subjects were equally distributed among cases and controls (for instance the study by Kuhen et al. [34])—which should result in a non-differential bias, thus biasing the association measure toward the null. However, omission of non-geocoded cases could distort associations in cohort study designs.

As in ecological studies, In individuals approaches including cohort or case–control studies, some misclassification of exposure may result from the used of census tract [35], postcode [49] or zip code [30] to define the location of maternal residence. In addition, census tracts or zip codes might not be valid measures of proximity because they vary considerably in size and are irregular in shape [28, 30, 35, 49].

In contrast with ecological approaches, in individuals approaches the authors may have additional information concerning residential history which may improve the exposure measure of women.

Exposure misclassification may occur where the birth certificate address does not reflect the mother’s true residence during the relevant window of fetal development [34, 45, 63, 64]. To assign exposure, many studies used maternal address at delivery rather than address around conception and during the first trimester, a period of particular relevance and vulnerability for fetal development. Few studies have considered exposure during pregnancy and the preconception period [32, 35, 40]. This can have a particular impact on studies exploring the risk of chromosomal and non-chromosomal congenital malformations, because organogenesis is essentially complete by the end of the first trimester of pregnancy, and most structural birth defects appear during this period [65]. In the case of non-chromosomal abnormalities (conotruncal heart defects, for example) the most critical period is during the first two months after conception [13, 49]. For chromosomal congenital abnormalities, the most appropriate residential exposure windows would include parental residences shortly before or at conception or even grand-maternal residences for some defects if the aberration occurred during maternal meiosis I [32, 33, 49, 66].

Misclassification of exposure may occur following changes in residence during the pregnancy [33, 49]. In general, studies are unable to take this limitation into account, due to a lack of information as to the pregnant women’s mobility [31, 34, 44]. Where available, estimation of residential mobility among pregnant women between conception and delivery differed between a Canadian [67] and a US study [64, 68, 69] and ranged from about 12% in the former to 32% in Texas [64]. However, of these, only 50% moved more than 1 km away [70] from the initial residence. This residential mobility may vary according to certain individual and contextual characteristics such as age, race, socioeconomic status and other factors. Higher mobility rates during pregnancy have been reported among whites, young mothers [69], less well-educated mothers [68], mothers with lower household income and higher pregnancy body mass index [67] and who lived near a hazardous waste site [71]. Some studies found that young mothers (<20 years) were more likely to move between conception and delivery than older mothers (>30 years) [33, 64]. This means that the exposure misclassification error due to using delivery address might be greater among younger mothers than among older ones, a phenomenon that might result in confounding—because age is also associated with the risk of poor pregnancy outcome.

This type of misclassification error may also tend to reduce the magnitude of estimated effects [68, 72]. Some studies estimate that this would lead, roughly, to a 10% underestimation of the true excess risk of congenital abnormality related to exposure during early pregnancy [73]. Where studies used case–control analysis, to the extent that residential proximity to a hazardous waste site was misclassified non-differentially among cases and controls, the results would have been biased toward the null [40]. Nevertheless, where authors have restricted their analysis to women who resided at the address noted on the vital record for at least 12 months before delivery or fetal death [74], only a slight increase in the OR was observed—still not significant [40]. On the other hand, in a study of women aged 35+, the association between maternal residence near industrial facilities during the periconceptional period and oral clefts was stronger than for maternal address at delivery [32].

##### Study design and risk estimate

In the ecological studies, the model relating risk to exposure to polluted site at individual level may differ to that at group level [75]. Thus, the relations observed between the variables at the group level (zip code, census block, village) cannot be directly transposed to the individual level [76, 77] even if several individual and ecological studies quantify the relation with the same association measure (the odds ratio or the relative risk). Whereas individuals’ studies are particularly advantageous to assess the risk around polluted sites. In addition, even among individuals’ studies, the different study designs (cohort or case–control) provide various quantitative risk estimates. Therefore, the analysis and the comparison of the impact of living around polluted sites are difficult because of the heterogeneity in study designs even if we know that under certain conditions, when the frequency of the health event is very low, as it the case in the present study (congenital malformation, LBW …) the OR gives a good estimate of the RR.

#### Assessment of the risk of pregnancy outcome around polluted sites

An array of factors will be evoked below. Firstly, the various confounding factors included in the individual studies lead difficult the comparisons between studies. Indeed, some studies did not use any covariates [31], while others adjusted only baby characteristics (sex, year of birth) without maternal characteristics [42–44, 50]. Others studies adjusted on baby and mother characteristics (maternal age, maternal education…) [33, 46], and less often on paternal characteristics [34, 40] while others selected four putative confounders, including baby and maternal characteristics and unhealthy behaviors among others (smoking and alcohol use) [29, 30, 32, 35, 36, 40, 55] or healthy behaviors (vitamin use) [35].

An absence of systematic adjustment for commonly known factors may affect the measure of association and thus the comparisons—for instance folic acid supplementation, which is known to decrease the risk of congenital malformation [78]. These risk factors tend to vary across the unit of analysis and if they are coincident with the exposure measures, then these spatial confounders will bias the results of the study. In ecological studies, if no individual’s data are available, choosing a spatial unit as small as possible will decrease the ecological bias because confounding may be less of a threat and more easily controlled in the analysis.

Secondly, the sample size of any statistical and epidemiological studies may affect the statistical power and led to show either (i) an absence of significant association of excess risk only by lack of power or; (ii) to show a significant association which is not validated because a low statistical power. In our review, we have various sample size from a very small sample (92 case of stillbirth in Bhopal study [48]) to large sample (136,821 cases of congenital malformation [11]) which can partially explain the diversity of findings even focusing on the study design; for instance, in ecological studies, some studies including 92 case of stillbirth [48] and 302 case of congenital malformations [48] did not reveal any significant excess risk among women living near petrochimical industries. While Cresswell, with 1508 case of congenital malformations, found a weak evidence of relation between prevalence of congenital malformations and residence near waste combustion plant [24]. However, some ecological studies included high sample size, including 136,821 cases of congenital malformation [11], 43,471 stillbirth [45], and found also significant weak associations.

In the individual studies, the authors investigated a more important sample size (7242 cases of fetal death [56], 6538 cases of PTB [55], except few studies which included only 63 cases of VLBW, 353 cases of LBW [29]. However, in case–control design study, several of them included no more than one control by case [12, 35, 54]; for instance: 7304 cases and 7834 control, [54], 507 cases and 517 control [35]. The number of controls per case is yet recognized to be a simple way to increase statistical power of studies.

While, other case–control included more than 3 control by cases [13, 32, 34, 39], for instance: 1244 case and 4368 control [13], 1289 case and 4965 control [32].

All the features of the studies describe above—such as study population, study design, sample size, the classification and definition of reproductive outcome, exposure assessment and confounding factors—could impact, independently or in combination, the quality of each study itself and also their comparison in our systematic review.

#### Future research

On the basis of this analysis of the limitations of the current body of research and of theoretical and methodological considerations, below we describe some suggestions for improvements to a research agenda.

#### Data accuracy

Lack of address-specific household data is a major impediment in assessing the health impact of residential proximity to polluted sites. Aggregated health data are most often not sufficiently fine-grained. While individual health data are more relevant, at least data collected at a fine resolution scale (such as census block) would improve the quality of the information. Neither is the spatial resolution of polluted site data generally precise. Site boundaries could be digitized instead of using simple points as location of exposure source, particularly when studying hazardous waste sites such as landfills.

#### Appropriate geostatistical approaches

Although environmental modeling is relatively cumbersome, labor-intensive, computer-intensive and requires extensive data input, it is still held out as the gold standard for environmental or health impact assessment. Some reliable alternative methods could be developed—preferably geostatistical approaches that are well-integrated or closely-coupled with GIS approaches—to estimate the contribution of various sources to total exposure, and to optimize exposure assessment. This calls for multidisciplinary teams having expertise in GIS, epidemiology, environmental science and statistical modeling.

In addition, in future studies, emphasis could be placed on the selection of focused-cluster test recognized to be more appropriate to spatial pattern of environmental exposure. More precisely, these spatial approaches have been designed and used to detect clusters reflecting a particular spatial pattern [79]; one that centers around the polluted sites and declines with increasing distance from the source.

Instead of investigating spatial data with common but rough approach (which is based on a circle of fixed radius around the point source with arbitrary size chosen by default and consist in the comparison of the frequency of cases inside with outside the buffer), the futures studies could use inferential method such as focused methods design to detect focused clustering around polluted site under the hypothesis that the risk of disease is high close to polluted site.

These spatial approaches use distance as a surrogate for exposure and assess whether cases are closer to the source than expected. Compared to other spatial methods, one advantage of these methods is that they address a specific hypothesis of concern and, because of their specificity, have increased sensitivity. Among these approaches, some authors proposed to use binary isotonic regression, known as Stone’s MLR test. One useful feature of isotonic regression is that the test result does not depend on whether one uses distance from the source of a measure of exposure for the analysis, as long as the exposure is decreasing with distance from the source [80].

#### Multi-hazard approaches

Most studies to date have looked at only one or two environmental hazards at a given point in time. Investigating the cumulative and synergistic impacts of the variety of chemical and non-chemical hazards and nuisances could help understand whether these impacts might be larger than currently acknowledged.

#### Enhanced exposure assessment

Because people do not spend their whole lives at home, future research should strive to incorporate residential mobility and daily commuting associated with occupational (or school) activities in exposure assessment. Further, the time window of exposure should be appropriate for each adverse reproductive outcome, from life-threatening teratogenic effects to weight or term at birth. This means that focusing on the preconceptional period, the first trimester of pregnancy or later, as most relevant.

#### Investigation of social inequalities

Today, there are significant gaps in our understanding of how disparities in exposure levels according to individual or contextual socioeconomic status (the ‘environmental justice’ issue) may or may not interact with other risk factors associated with social deprivation, such as poor access to health care, a low level of physical activity or high prevalence of smoking. Future studies on reproductive outcome in relation to proximity to polluted sites might accommodate relevant study designs and data analyses approaches to explore the assumption that social deprivation might represent an effect modifier for exposure to hazardous substances in such settings.

## Conclusions and public policy perspectives

Despite improvements to the control of emission measurements in industrialized countries since the 1990s, (‘Superfund’ in the USA, ‘IPCC’ in the European Union and similar provision), there are suggestive evidences that residential proximity to polluted sites (including landfills, hazardous waste sites and industrial facilities) might contribute to adverse reproductive outcomes, especially congenital malformation and low birth weight—However, no studies show significant excess risk of mortality including fetal death, neonatal or infant mortality and stillbirth. In order to focus on preventive actions and provide useful tools, we need to better understand and interpret our findings, considering weaknesses which could affect the strength of associations, yield limitations in comparisons or impede the formulation of accurate conclusions. However, our study should trigger hypotheses which would recommend strengthening the rules governing industrial emissions and industrial waste management, and reinforce land-use planning with regard to the most polluted sites.
